# Using eQTL Mendelian randomization and transcriptomic analysis to identify the relationship between ion channel genes and intracranial aneurysmal subarachnoid hemorrhage

**DOI:** 10.1097/MD.0000000000042457

**Published:** 2025-05-16

**Authors:** Jing Wang, Bowang Chen, Qiang Meng, Feng Qu, Zhen Ma

**Affiliations:** a Department of Intensive Care Unit, Jining No. 1 People’s Hospital, Shandong, China.

**Keywords:** aneurysmal subarachnoid hemorrhage, expression quantitative trait loci, genome-wide association analysis, ion channel genes, Mendelian randomization, transcriptome

## Abstract

Aneurysmal subarachnoid hemorrhage (aSAH) is a complex condition associated with high disability and mortality rates, leading to poor clinical outcomes. Previous observational studies have suggested a link between ion channel genes and aSAH, but the causal relationship remains uncertain. This study utilized Mendelian randomization (MR) to explore the causal association between ion channel genes and aSAH, employing 5 MR methods: inverse variance weighted (IVW), MR-Egger, maximum likelihood, weighted median, and weighted mode. If results from these methods are inconclusive, IVW will be prioritized as the primary outcome. Additionally, MR-Egger, MR-PRESSO, and Cochrane *Q* tests were conducted to assess heterogeneity and pleiotropy. The stability of MR findings was evaluated using the leave-one-out approach; Bonferroni correction tested the strength of the causal relationship between exposure and outcome. The MR analysis revealed that CACNA2D3 was positively correlated with aSAH (OR 1.245; 95% confidence intervals [CI] 1.008–1.537; *P* = .042), while ANO6 showed a negative correlation (OR 0.728; 95% CI 0.533–0.993; *P* = .045). Our findings indicate that increased expression of CACNA2D3 promotes aSAH whereas elevated levels of ANO6 inhibit it. Transcriptome data from intracranial aneurysm samples confirmed significant differential expression of CACNA2D3 and ANO6 between ruptured and unruptured groups. CACNA2D3 being higher in ruptured cases while ANO6 was more expressed in unruptured ones. Furthermore, GeneMANIA analysis along with functional enrichment provided insights into risk factors for aSAH. Through MR analysis, we established a causal link between ion channel genes and aSAH, which helps to better understand the pathogenesis of aSAH and provide new therapeutic targets.

## 1. Introduction

Intracranial aneurysm (IA) is characterized by the localized pathological dilation of the intracranial artery wall, resulting in a pouch-like protrusion, with an estimated prevalence of approximately 3.2% in the general population.^[1]^ Approximately 80% of subarachnoid hemorrhage cases are attributed to IA rupture, referred to as aneurysmal subarachnoid hemorrhage (aSAH).^[2]^ A systematic review and meta-analysis encompassing 75 studies involving 8176 patients revealed that the crude global incidence rate of aSAH declined from 10.2 per 100,000 individuals annually in 1980 to 6.1 per 100,000 individuals annually in 2010; however, significant variations were observed based on region, age, and sex.^[3]^ Despite this reduction in incidence rates, aSAH continues to be a catastrophic cerebrovascular condition with mortality rates reaching up to 50% and morbidity rates as high as 70%.^[4,5]^ In comparison to ischemic stroke patients, those affected by aSAH tend to be approximately 20 years younger on average; this demographic difference contributes to prolonged chronic phases and disability durations while necessitating greater allocation of social resources.^[6]^ Although advancements in medical technology and innovations such as neurointerventional techniques have markedly improved treatment outcomes for IAs, the mortality and disability rates associated with aSAH remain alarmingly elevated. Therefore, it is imperative to further investigate and reassess the pathophysiological mechanisms underlying aSAH along with its potential contributing factors.

Ion channels are manifested in virtually all vascular cell types, encompassing endothelial cells, smooth muscle cells, and fibroblasts.^[7–9]^ These ion channels exert pivotal functions in a plethora of physiological regulatory processes, such as membrane potential maintenance, signal transduction, hemodynamics, and vascular contractility.^[10,11]^ The dysregulation of the expression and functionality of vascular cell ion channels constitutes a salient mechanism underlying the pathogenesis and progression of cardiovascular disorders like hypertension, coronary artery disease, and atherosclerosis.^[12]^ Contemporary research indicates that vascular remodeling driven by chronic inflammatory responses resulting from aberrant blood flow is of paramount significance in IA formation, expansion, and rupture.^[13]^ Distinct classes of ion channels within vascular cells are intimately associated with the processes of vascular remodeling and fibrosis.^[14]^ Animal studies have demonstrated alterations in ion channel activity in cerebral arterioles and basilar arteries subsequent to aSAH.^[15,16]^ Furthermore, in addition to alterations in gene expression associated with voltage-gated calcium channels (VGCCs) following aSAH, vasoactive substances released into the cerebrospinal fluid may also enhance the activity of preexisting VGCCs in the smooth muscle cells of cerebral arteries.^[17]^ Despite the copious evidence suggesting an association between ion channels and aSAH, the majority of investigations remain observational, circumscribed by sample size limitations and confounding variables. A robust correlation exists between variations in ion channel expression and their corresponding genes. Therefore, elucidating the causal relationship between ion channel genes and aSAH may facilitate the unearthing of novel biological mechanisms associated with this condition.

Mendelian randomization (MR) is a methodological approach employed to infer causal relationships between exposure and outcome.^[18]^ In this study, we integrated expression quantitative trait loci (eQTL) eQTL data for ion channel genes with summary data from genome-wide association studies (GWAS) pertaining to aSAH in order to investigate the causal relationship between ion channel gene expression and the complex traits associated with aSAH. The findings of this study provide novel insights into understanding the interplay between ion channel genes and aSAH while contributing to an enhanced comprehension of its pathogenic mechanisms.

## 2. Methods

### 2.1. Study design

**Figure 1.**
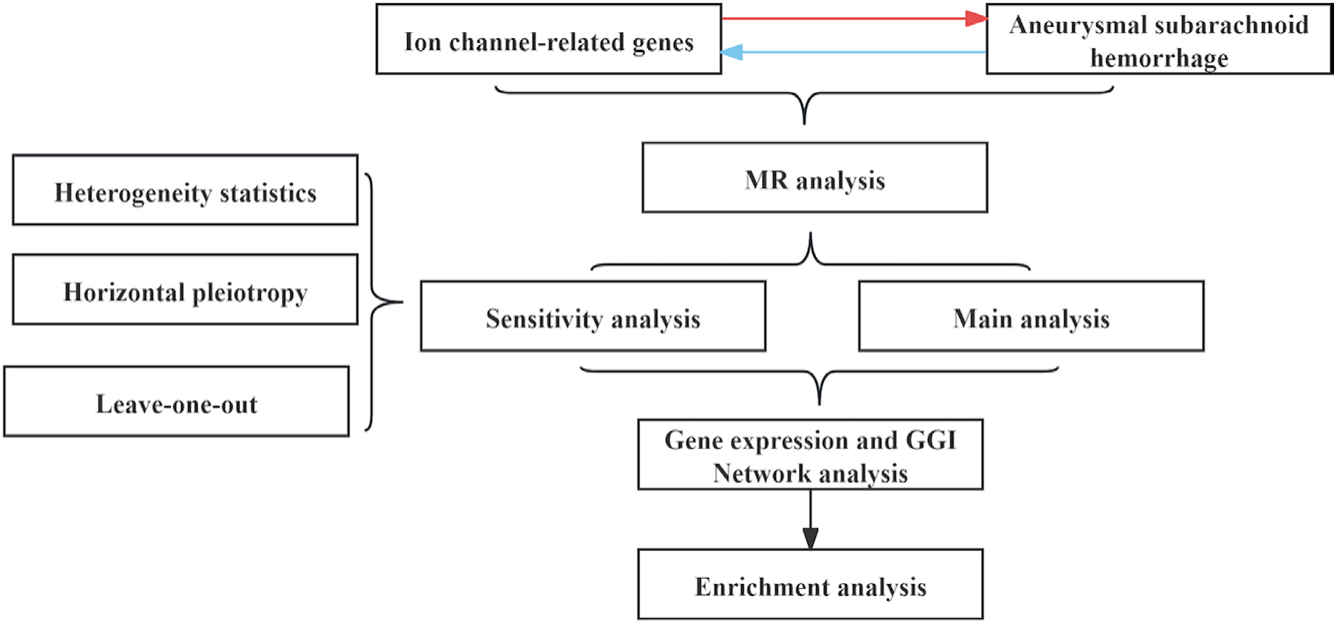
Flowchart of this study. The study design integrates eQTL data, Mendelian randomization, transcriptomic validation, and functional enrichment analysis to investigate the causal role of ion channel genes in aSAH. aSAH = aneurysmal subarachnoid hemorrhage, eQTL = expression quantitative trait loci.

This study utilized eQTL data from 172 ion channel genes and pooled summary data from a GWAS of aSAH to perform a bivariate MR analysis. We selected single nucleotide polymorphisms (SNPs) that exhibited significant associations with exposure factors (*P*-value threshold < 5 × 10^–6^) as instrumental variables (IVs), employing inverse variance weighted (IVW) as the primary analytical method. Additionally, sensitivity analyses and heterogeneity tests were conducted to ensure the robustness of our findings. The differentially expressed ion channel genes identified through MR were validated using transcriptomic data, followed by GeneMANIA and functional enrichment assessments, as illustrated in Figure 1.

### 2.2. Data sources and extracts

In this study, 330 ion channel genes were obtained from the Human Genome Organisation Gene Nomenclature Committee (HGNC) database (see Table S1, Supplemental digital content, https://links.lww.com/MD/O910 online). The HGNC is jointly funded by the UK Medical Research Council and the US National Institutes of Health, providing a unique identifier for each gene.^[19]^ Utilizing the R package TwoSampleMR, we retrieved GWAS IDs for ion channel genes from the IEU OpenGWAS database. We identified 172 ion channel genes with eQTL present in the GWAS summary data (see Table S2, Supplemental Digital Content, https://links.lww.com/MD/O911 online), which served as exposure factors. aSAH was designated as the outcome variable in this analysis. The summary data were derived from the cohort characterized by Bakker, which comprises 23 distinct cohorts of individuals of European ancestry, encompassing a total of 5425 cases of IA rupture and 71,934 control subjects.^[20]^

Furthermore, transcriptomic data related to aSAH were sourced from GSE13353 within the GEO database, encompassing tissue samples and clinical information from 11 ruptured IAs and 8 unruptured IAs among individuals of European ancestry.^[21]^ These data were utilized for differential expression analysis.

### 2.3. Instrumental variables and data harmonization

Our study adhered to the 3 fundamental assumptions of MR analysis. Firstly, the SNPs utilized in MR analysis must be closely associated with ion channel genes. Selecting SNPs that are linked to the eQTL of ion channel genes as IVs allows for inferring a direct causal relationship between gene expression and outcomes, thereby facilitating the identification of novel risk genes and pathogenic mechanisms, as well as advancing the development of gene-targeted therapeutic strategies. In this investigation, to ensure an adequate number of IVs were included, we selected SNPs with *P*-value threshold < 5 × 10^–6^ for analysis. To effectively mitigate bias introduced by weak IVs, we chose IVs with an *F* statistic >10 (formula: *F* = *R*^2^(n − 2)/(1 − *R*^2^),^[22]^ where *R*^2^ denotes the coefficient of determination and n represents the sample size. Secondly, it is essential that the selected IVs satisfy independence criteria. We established a linkage disequilibrium threshold for SNP pairs at *r*^2^= 0.001 and kb = 10,000 to eliminate potential confounding effects from linkage disequilibrium while maintaining independence among selected IVs. Thirdly, these IVs should not exhibit any association with the outcome variable; thus, we excluded those related to aSAH at *P*-value < .05. During this process, palindromic SNPs were removed through coordinated effects while correcting allelic SNP discrepancies. These selection criteria ensured the robustness and validity of our research findings.

### 2.4. Mendelian randomization analysis

In this study, we employed 5 methodologies for the 2-sample MR analysis: the IVW method, MR-Egger regression, weighted median estimation, maximum likelihood estimation, and weighted mode analysis. Among these methods, IVW served as the primary analytical approach. All MR analyses were conducted using the 2-Sample MR R package.^[23]^ Within the context of MR-Egger regression, the intercept was utilized to detect pleiotropy; Cochran *Q* statistic (*Q*) and Rucker *Q* statistic (*Q*’) were employed to assess heterogeneity in both IVW and MR-Egger estimates.^[24]^ The difference between these 2 *Q*–*Q*’ can be used to evaluate horizontal pleiotropy within the MR estimates. When both the *Q* statistic and its corresponding *P*-value are <.05, it indicates a significant presence of directional pleiotropy.^[25]^ Furthermore, a leave-one-out sensitivity analysis was performed to ascertain the influence of potentially significant SNPs, while Bonferroni correction was applied to adjust for multiple testing.

### 2.5. Expression analysis of candidate ion genes, GeneMANIA and functional enrichment analysis

In this study, we integrated the GSE13353 dataset and employed the R packages ggpubr and wilcox.test to compare the expression profiles of candidate ion channel genes between ruptured and unruptured IA groups. *P*-value < .05 was considered indicative of significant differential expression between the 2 groups. The genes that were successfully validated are classified as key ion channel genes and will be utilized for subsequent GeneMANIA and functional enrichment analyses.

## 3. Results

### 3.1. Selection of instrumental variables

Following the removal of linkage disequilibrium, this study extracted comprehensive SNPs data from the eQTL associated with ion channel genes, including beta coefficients, standard errors, *P*-values, effect alleles, and alternative alleles. Additionally, we calculated the *F* statistic, and all SNPs demonstrated *F*-values>10, indicating the absence of weak IVs in this analysis.

### 3.2. The results of 2-sample Mendelian randomization

**Figure 2.**
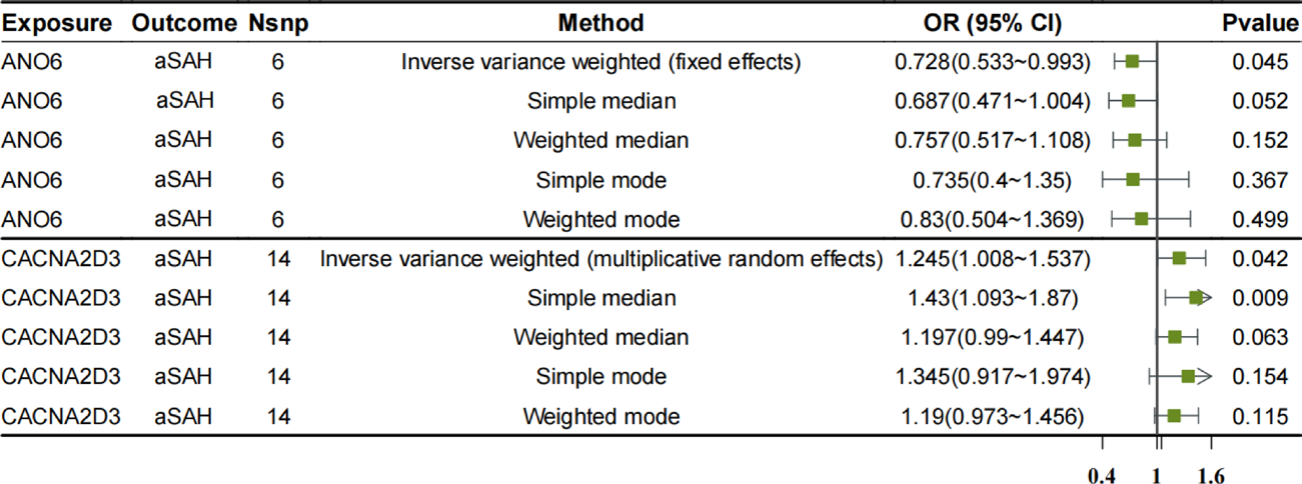
The forest plot for the MR analysis features. Results of MR analysis for CACNA2D3 and ANO6, showing odds ratios (OR) and 95% CI. CI = confidence intervals, MR = Mendelian randomization.

**Figure 3.**
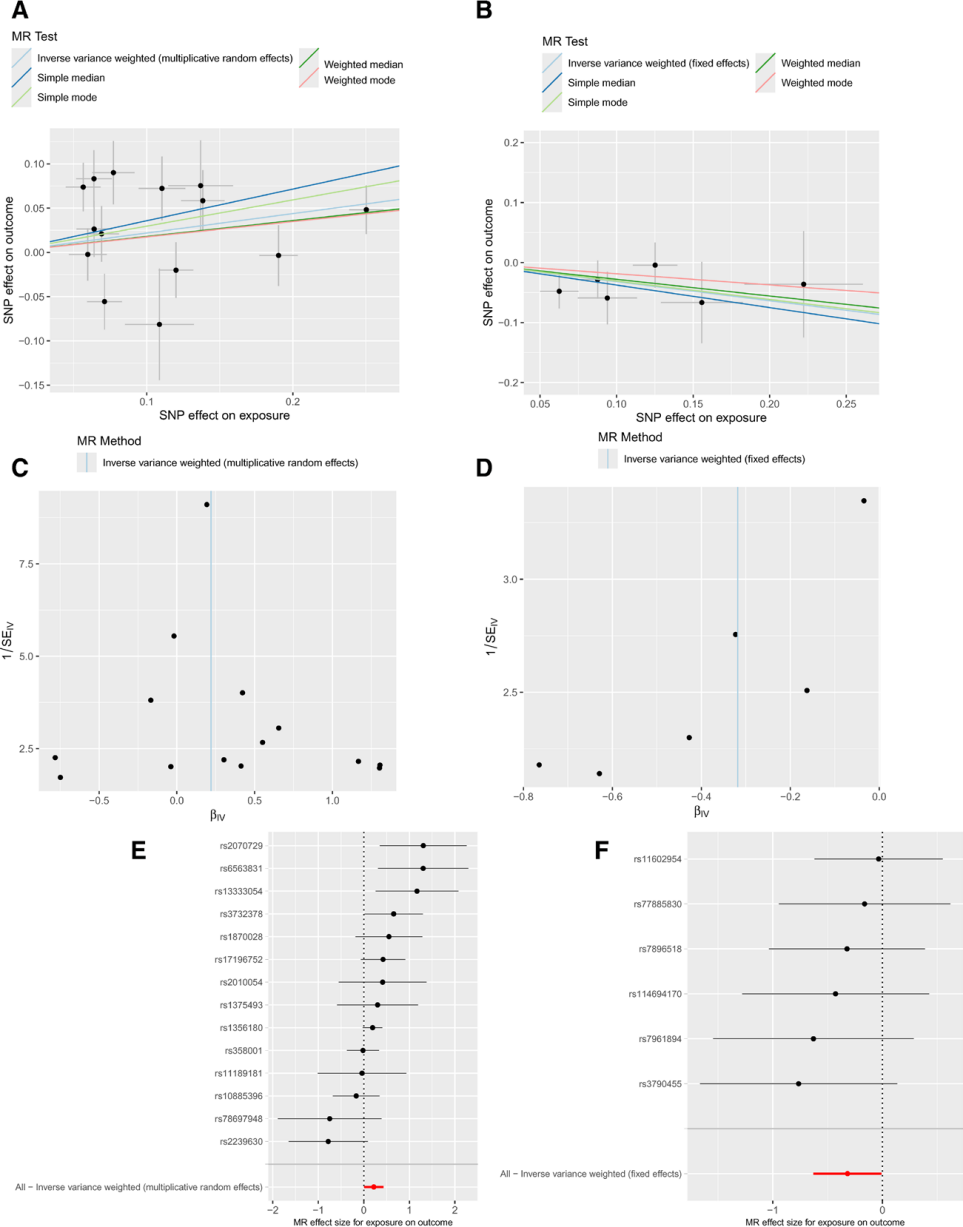
Mendelian randomization results for ion channel genes and aSAH. (A and B) Scatter plots of genetic associations; (C and D) funnel plots assessing heterogeneity; (E and F) forest plots summarizing effect estimates. aSAH = aneurysmal subarachnoid hemorrhage.

In this study, ion channel genes were designated as the exposure factors, with aSAH defined as the outcome variable. The MR analysis was conducted using 5 distinct algorithms (see Table S3, Supplementary Digital content, https://links.lww.com/MD/O912). The ion channel genes CACNA2D3 and ANO6 exhibited *P*-values＜.05, indicating that these 2 genes represent significant genetic risk factors for aSAH. Specifically, 14 single nucleotide SNPs were utilized as IVs for the MR analysis of CACNA2D3, whereas 6 SNPs were employed as IVs for ANO6. The application of the IVW method demonstrated that an increase in CACNA2D3 was significantly positively correlated with an elevated risk of aSAH (OR = 1.245; 95% confidence intervals (CI): 1.008–1.537; *P* = .042), whereas an increase in ANO6 was significantly negatively correlated with a decreased risk of aSAH (OR = 0.728; 95% CI: 0.533–0.993; *P* = .045), as illustrated in Figure 2. When evaluating the correlation between exposure factors and outcomes, the presence of an intercept indicates that potential confounding factors may be present; however, the IVW analysis revealed that this intercept was minimal, suggesting a limited influence of confounders on the reliability of our findings. Furthermore, the slope associated with CACNA2D3 was positive, indicating its role as a risk factor, whereas ANO6 is considered protective in nature. In terms of randomness assessment, the samples demonstrated an approximately symmetrical distribution on both sides of the IVW line, consistent with Mendel second law of inheritance. Consequently, the estimated diagnostic efficacy at each SNP site further corroborated that CACNA2D3 functions as a risk factor while ANO6 acts as a protective factor against aSAH. Therefore, this study employed 5 MR models to evaluate stable causal associations between CACNA2D3 and ANO6 in relation to aSAH, as illustrated in Figure 3.

### 3.3. The assessment of heterogeneity and sensitivity in Mendelian randomization

**Figure 4.**
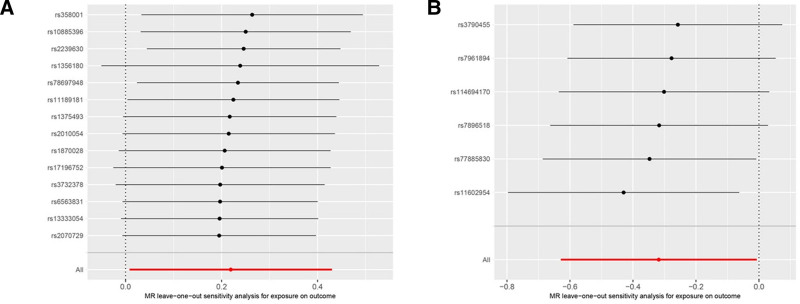
Sensitivity analysis of Mendelian randomization. Leave-one-out plots for CACNA2D3 (A) and ANO6 (B) demonstrate the robustness of MR results. MR = Mendelian randomization.

The results of the heterogeneity test revealed that the Q_pval for CACNA2D3 was <.05, indicating the presence of heterogeneity; conversely, the Q_pval for ANO6 was >.05, suggesting an absence of heterogeneity. (see Table S4, Supplemental Digital Content, https://links.lww.com/MD/O913 online). Accordingly, a random-effects IVW model was applied for CACNA2D3 in the MR analysis, whereas a fixed-effects IVW model was employed for ANO6. Both horizontal pleiotropy tests and MR-PRESSO analyses did not reveal any evidence of horizontal pleiotropy (see Table S5, Supplemental Digital Content, https://links.lww.com/MD/O914 online). Moreover, “Leave-One-Out” sensitivity and heterogeneity analyses revealed that the exclusion of individual IV did not result in any abnormal findings in the MR analysis related to these ion channel genes and aSAH. Furthermore, no instrumental variable exerted a significant influence on the MR outcomes, as depicted in Figure 4. The Steiger direction test was performed to validate the directional causality between exposure and outcome. The directional test *P*-value < .05 suggests that the identified directionality is valid. (see Table S6, Supplemental Digital content, https://links.lww.com/MD/O915 online).

### 3.4. Analysis of the expression profiles of CACNA2D3 and ANO6 in the walls of IA

**Figure 5.**
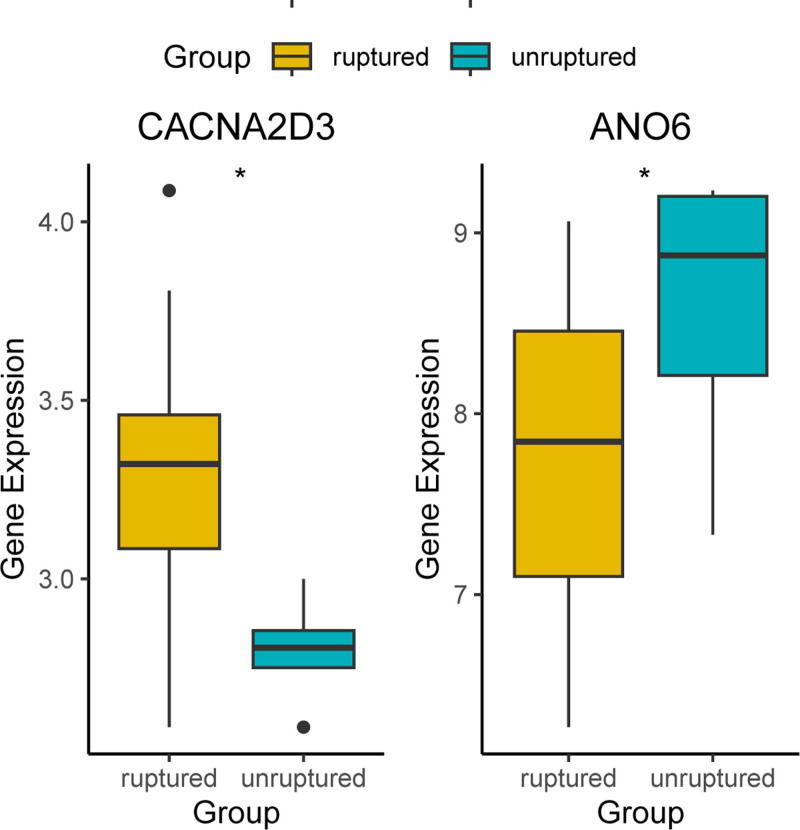
Expression distribution of CACNA2D3 and ANO6 between ruptured and unruptured (IA groups. Violin plots show significant differential expression (Wilcoxon test, *P* < .05). IA = intracranial aneurysm.

A significant difference was observed in the expression levels of the ion channel genes CACNA2D3 and ANO6 between ruptured and unruptured intracranial IA groups (*P*-values < .05). Specifically, CACNA2D3 exhibited elevated expression in the ruptured group, while ANO6 demonstrated higher expression levels in the unruptured group，as illustrated in Figure 5.

### 3.5. GeneMANIA analysis of CACNA2D3 and ANO6

**Figure 6.**
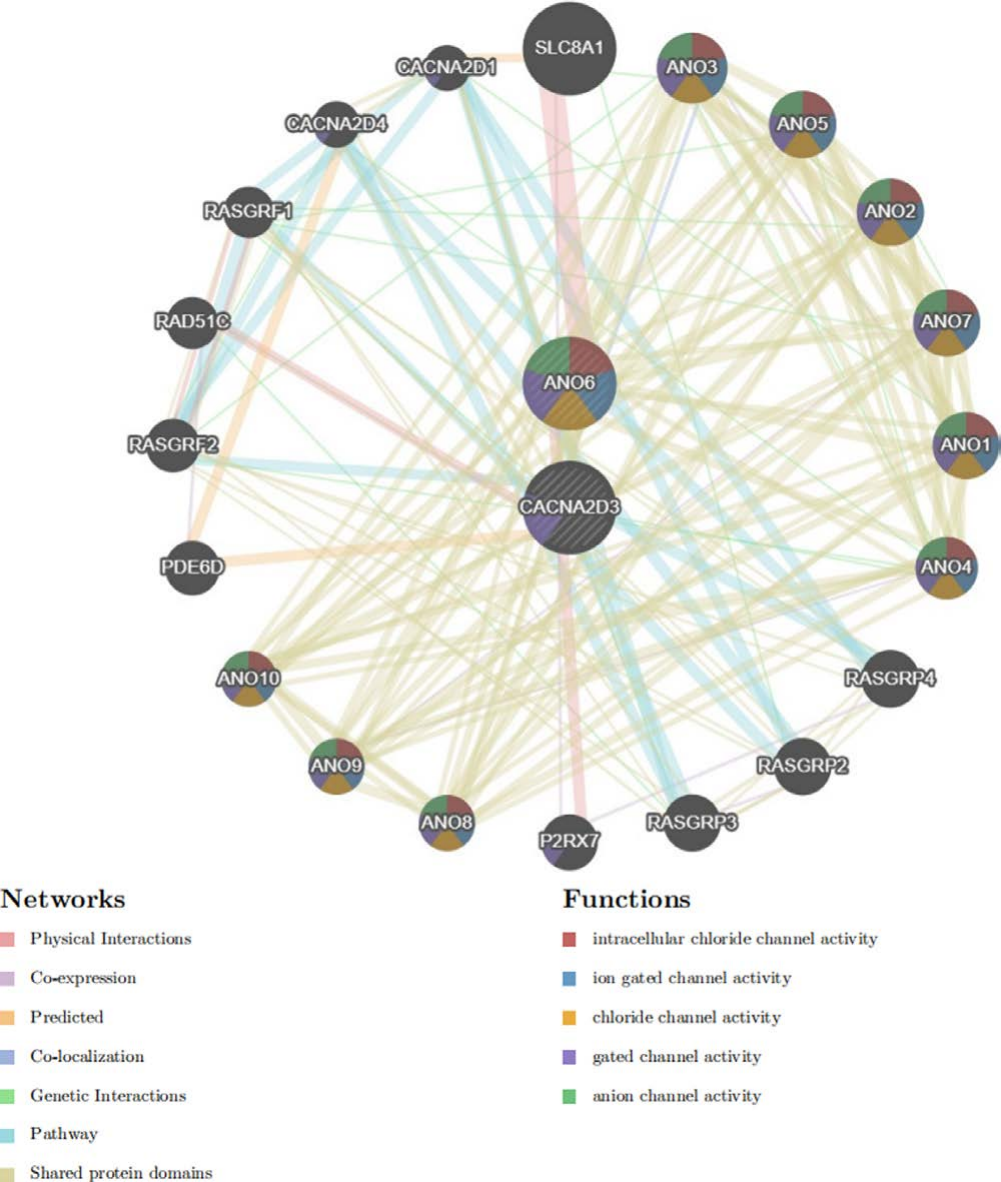
Gene interaction network constructed by GeneMANIA. Functional associations of CACNA2D3 and ANO6 with related ion channel activity pathways.

With respect to CACNA2D3 and ANO6, we identified 20 hub genes associated with these targets. ANO6 is linked to intracellular chloride channel activity, ion-gated channel activity, chloride channel activity, gated channel activity, and anion channel activity; in contrast, CACNA2D3 is primarily associated with gated channel activity. Detailed information can be found in Figure 6.

### 3.6. Gene set enrichment analysis (GSEA) of CACNA2D3 and ANO6

**Figure 7.**
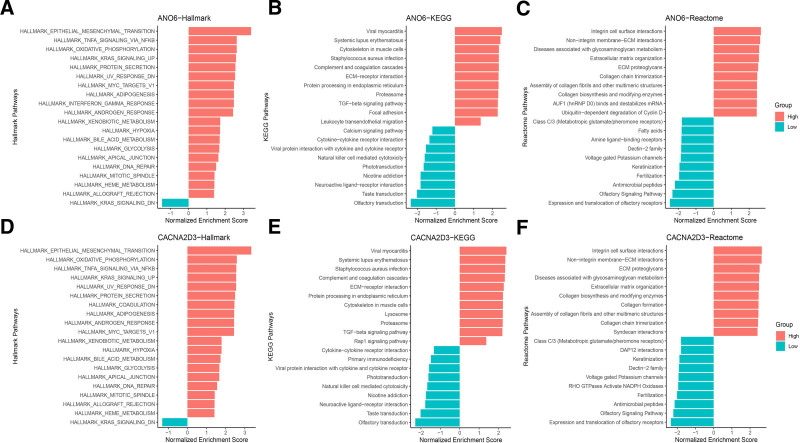
Pathway enrichment analysis of ANO6 and CACNA2D3. Hallmark, KEGG, and reactome pathways highlight roles in ECM remodeling, immune response, and calcium signaling. ECM = extracellular matrix, KEGG = Kyoto Encyclopedia of Genes and Genomes.

In the enrichment analyses conducted for Kyoto Encyclopedia of Genes and Genomes, Hallmark, and Reactome, CACNA2D3 was associated with 139, 41, and 496 pathways, respectively. The results of the GSEA indicated that CACNA2D3 is linked to several pathways including influence extracellular matrix (ECM)–receptor interaction, transforming growth factor (TGF)-β signaling pathway, oxidative phosphorylation, non-integrin membrane-ECM interactions, and extracellular matrix organization. Additionally, in the aforementioned 3 enrichment methods, gene ANO6 was associated with 140, 41, and 464 pathways, respectively. The GSEA outcomes demonstrate that ANO6 is correlated with pathways such as cytokine-cytokine receptor interactions, TNFα/nuclear factor kappa B, ECM proteoglycans, and integrin cell surface interactions, as illustrated in Figure 7.

## 4. Discussion

To our knowledge, this study represents the inaugural application of a multi-omics integration approach to identify potential ion channel genes in blood and IA wall tissues, along with elucidating their underlying mechanisms in aSAH. The prevention and management of aSAH pose significant challenges, primarily due to the ambiguous pathophysiological mechanisms associated with the formation and rupture of IA. A causal relationship has been established between the ion channel genes CACNA2D3 and ANO6 and aSAH, wherein increased expression of CACNA2D3 is positively correlated with an elevated risk of aSAH, while heightened levels of ANO6 are inversely associated with a reduced risk. Furthermore, this study substantiates the absence of reverse causality. Additionally, transcriptome data reveal that CACNA2D3 is markedly upregulated in the walls of ruptured IAs, whereas ANO6 exhibits significantly higher expression in unruptured aneurysm walls. Collectively, these findings suggest that alterations in the expression profiles of key risk genes may play a pivotal role in the etiology of aSAH.

IA is a pathological outward bulging of the intracranial artery, which may be associated with localized thinning of the arterial wall and predisposes it to rupture.^[26]^ The primary mechanisms underlying the formation and progression of IA encompass: endothelial injury and degradation of the elastic layer; recruitment and infiltration of inflammatory cells; and chronic remodeling of the vascular wall.^[27]^ Research has shown that alterations in ion channel configuration within endothelial cells can result in functional impairments.^[28]^ Pathological examinations of human IA walls reveal significant inflammatory infiltration, predominantly characterized by the presence of macrophages and T cells.^[29,30]^ Ion channels are highly expressed in immune cells and play a critical role in maintaining immune activity, regulating lymphocyte development, and modulating immune responses.^[31]^ Furthermore, the expression levels of different types of ion channels in vascular smooth muscle cells are closely linked to processes associated with vascular remodeling and fibrosis.^[15]^ Consequently, it is evident that ion channels are intricately linked to both the development of IA and aSAH.

GeneMANIA analysis indicates that CACNA2D3 is closely associated with the regulation of ion channels. VGCCs are multi-subunit protein complexes composed of an α1 subunit and 3 regulatory subunits (α2δ, β, and γ).^[32,33]^ CACNA2D3 specifically encodes the α2δ3 subunit.^[34]^ Research has demonstrated that as a regulatory subunit, α2δ3 facilitates calcium ion translocation across the membrane via VGCCs.^[35]^ During cell membrane depolarization, the opening of VGCCs permits calcium ions to enter cells, thereby influencing cellular excitability, contractility, and other physiological processes. Current studies indicate that mutations, abnormal expression levels, or functional impairments in the CACNA2D3 gene are closely linked to various diseases including neurological disorders,^[36]^ cancers,^[37]^ and type 2 diabetes.^[38]^ Furthermore, GeneMANIA analysis reveals that ANO6 activity primarily involves chloride ion transport through gating mechanisms. ANO6 (also known as TMEM16F) encodes a calcium-dependent chloride channel belonging to the TMEM16 family.^[39]^ The protein product (ANO6 or TMEM16F) promotes chloride efflux and is intricately connected to calcium signaling pathways.^[40]^ Abnormal expression and mutations in the ANO6 gene have been significantly associated with pathogenic mechanisms underlying several diseases such as Scott syndrome,^[41]^ bone formation disorders,^[42]^ and Alzheimer disease.^[43]^ Functional enrichment analysis revealed that pathways associated with CACNA2D3, including cell adhesion receptor interactions, TGF-β signaling pathway, non-integrin membrane-extracellular matrix interactions, and ECM remodeling, emphasize the critical relationships between cells and their microenvironment, which influence tissue architecture, cell migration, and fibrosis among other processes. Conversely, the oxidative phosphorylation pathway encompasses fundamental processes such as energy metabolism, signal transduction, and cellular fate determination. Furthermore, the 4 pathways enriched by ANO6 primarily regulate cellular functions through the modulation of intracellular signaling mechanisms; these pathways are predominantly implicated in immune responses, inflammation, and cell proliferation. This finding carries significant academic implications.

The pathological examination of the wall of IAs reveals that unruptured aneurysms are characterized by minor structural changes and a reduced number of inflammatory cells, predominantly consisting of M1 macrophages. In contrast, ruptured IAs exhibit significant structural alterations, a marked increase in inflammatory cell populations, and predominance of M2 macrophages.^[44]^ Research demonstrates that M1 macrophages are predominantly characterized by pro-inflammatory properties, whereas M2 macrophages are primarily associated with anti-inflammatory responses and tissue repair mechanisms.^[45]^ The enriched pathways of the CACNA2D3 are linked to M2 macrophages, while the functional enrichment analysis of ANO6 shows a stronger association with M1 macrophages. Therefore, we hypothesize that changes in the expression of CACNA2D3 and ANO6 genes may regulate the polarization of macrophages, thereby affecting the development and rupture of IA, leading to aSAH.

The upregulation of CACNA2D3 expression facilitates the influx of Ca^2+^ into endothelial cells, thereby disrupting their calcium homeostasis and resulting in intracellular calcium overload. This overload triggers mitochondrial dysfunction via the oxidative phosphorylation pathway, subsequently promoting apoptosis in endothelial cells.^[46]^ The apoptosis of endothelial cells induces surrounding active and apoptotic cells to produce TGF-β.^[47]^ Research has demonstrated that endothelial cell apoptosis can initiate monocyte recruitment, myofibroblast differentiation, and accumulation of adjacent tissues. Upon stimulation by TGF-β, monocytes bind this factor to its receptor, activating the downstream Smad signaling pathway. The Smad protein complex then translocates to the nucleus where it regulates the expression of various genes and promotes differentiation towards an M2 phenotype.^[48]^ M2 macrophages ECM–receptor interactions as well as ECM-related pathways through the secretion of diverse chemokines and proteases, thus regulating ECM degradation and remodeling processes.^[49,50]^ Furthermore, M2 macrophages interact with ECM components such as hyaluronic acid by expressing receptors like CD44, which affects their adhesive properties and migratory capabilities.^[51]^ The expression of the ANO6 gene is downregulated in aSAH, whereas validation of group data indicates an upregulation of this gene in unruptured IA. This gene encodes Anoctamin-6, a Ca^2+^-activated ion channel that also exhibits superoxide dismutase activity and facilitates the translocation of phosphatidylserine from the inner to the outer leaflet.^[52]^ The exposure of phosphatidylserine can activate ADAM17 (TNF-α convertase), leading to the release or dissociation of pro-inflammatory mediators such as TNF-α and interleukin-6 (IL-6).^[53]^ Upon binding to its receptor, TNF-α activates the nuclear factor kappa B signaling pathway, which induces the expression of pro-inflammatory cytokines and promotes macrophage polarization towards the M1 phenotype.^[54]^ M1 macrophages are capable of releasing substantial amounts of pro-inflammatory cytokines, thereby exacerbating systemic inflammatory responses. Furthermore, pro-inflammatory mediators like TNF-α and IL-6 can modulate proteoglycan metabolism through ECM-related pathways, playing critical roles in inflammation and tissue remodeling processes.^[55]^ Additionally, TNF-α and IL-6 can initiate integrin-mediated interactions by regulating integrin expression and activation pathways, facilitating recruitment and localization of various immune cell types within the immune system.^[56]^ This presentation elucidates how alterations in the expression of CACNA2D3 and ANO6 genes can drive macrophage differentiation into M1 or M2 phenotypes, while also discussing the potential mechanisms involved in the formation and rupture of IA. Advancements in imaging technology, coupled with an increasingly aging population, have led to a steady rise in the detection rate of asymptomatic unruptured IA, thereby presenting clinicians with a clinical dilemma: whether to pursue preventive treatment or adopt a watchful waiting strategy. This research identifies a novel potential biomarker for high-risk unruptured IA, facilitating early intervention aimed at reducing the incidence of aSAH.

Several constraints need to be resolved in the present research. Firstly, our research only incorporated 172 ion channel genes and their eQTLs. Future research should incorporate additional ion channel genes, which is essential for a more thorough understanding of the risk factors and underlying mechanisms of aSAH. Secondly, MR analyses can ascertain whether the observed correlations have causal relationships based on genetic evidence, which is regarded as a causal assumption. To establish the precise causal relationship between ion channel genes and aSAH, further laboratory and clinical investigations are typically required to uncover the potential biological mechanisms. While our study examined the mechanisms underlying the causal relationship between ion channel genes and aSAH, additional clinical trials and mechanistic investigations are necessary for confirmation. Thirdly, the transcriptomic validation cohort included 11 ruptured and 8 unruptured aneurysms, which may limit the generalizability of our findings. While the differential expression of CACNA2D3 and ANO6 was statistically significant, larger cohorts are needed to confirm these results and reduce potential biases inherent to small sample sizes.

In summary, our research offers MR evidence that supports the causal involvement of the ion channel genes CACNA2D3 and ANO6 in aSAH. We aim to achieve a deeper understanding of the mechanisms of the complex disease aSAH and to identify potential risk factors.

## Acknowledgments

The author would like to express his sincere gratitude to GEO database and GWAS database for sharing their data.

## Author contributions

**Conceptualization:** Jing Wang, Bowang Chen, Zhen Ma.

**Data curation:** Jing Wang, Bowang Chen, Zhen Ma.

**Formal analysis:** Jing Wang, Bowang Chen, Feng Qu, Zhen Ma.

**Funding acquisition:** Jing Wang, Feng Qu, Zhen Ma.

**Investigation:** Bowang Chen, Feng Qu, Zhen Ma.

**Methodology:** Qiang Meng, Zhen Ma.

**Project administration:** Jing Wang, Bowang Chen, Qiang Meng, Feng Qu, Zhen Ma.

**Resources:** Jing Wang, Bowang Chen, Qiang Meng, Feng Qu, Zhen Ma.

**Software:** Bowang Chen, Qiang Meng, Zhen Ma.

**Supervision:** Jing Wang, Bowang Chen, Qiang Meng, Zhen Ma.

**Validation:** Qiang Meng, Zhen Ma.

**Visualization:** Qiang Meng, Zhen Ma.

**Writing – original draft:** Jing Wang, Bowang Chen, Zhen Ma.

**Writing – review & editing:** Jing Wang, Zhen Ma.

## Supplementary Material
