# Supplementary material for: Using eQTL Mendelian randomization and transcriptomic analysis to identify the relationship between ion channel genes and intracranial aneurysmal subarachnoid hemorrhage
Source: Medicine (Baltimore). 2025 May 16;104(20):e42457. doi: 10.1097/MD.0000000000042457 (PMC12091597; doi:10.1097/MD.0000000000042457)
Supplement: Supplementary file 1 [file medi-104-e42457-s001.docx]

| **Table S1** **Ion Channel Genes from the HGNC Database** | | | | | | | | | | | | |
| --- | --- | --- | --- | --- | --- | --- | --- | --- | --- | --- | --- | --- |
| HGNC ID | Approved symbol | Approved name | Status | Locus type | Previous symbols | Alias symbols | Chromosome | NCBI Gene ID | Ensembl gene ID | Vega gene ID | Group ID | Group name |
| HGNC:5297 | HTR3A | 5-hydroxytryptamine receptor 3A | Approved | gene with protein product | HTR3 | 5-HT3R, 5-HT3A | 11q23.2 | 3359 | ENSG00000166736 | OTTHUMG00000160494 | 172 | 5-hydroxytryptamine receptors, ionotropic |
| HGNC:5298 | HTR3B | 5-hydroxytryptamine receptor 3B | Approved | gene with protein product | | 5-HT3B | 11q23.2 | 9177 | ENSG00000149305 | OTTHUMG00000168210 | 172 | 5-hydroxytryptamine receptors, ionotropic |
| HGNC:24003 | HTR3C | 5-hydroxytryptamine receptor 3C | Approved | gene with protein product | | | 3q27.1 | 170572 | ENSG00000178084 | OTTHUMG00000156862 | 172 | 5-hydroxytryptamine receptors, ionotropic |
| HGNC:24004 | HTR3D | 5-hydroxytryptamine receptor 3D | Approved | gene with protein product | | | 3q27.1 | 200909 | ENSG00000186090 | OTTHUMG00000156858 | 172 | 5-hydroxytryptamine receptors, ionotropic |
| HGNC:24005 | HTR3E | 5-hydroxytryptamine receptor 3E | Approved | gene with protein product | | | 3q27.1 | 285242 | ENSG00000186038 | OTTHUMG00000156857 | 172 | 5-hydroxytryptamine receptors, ionotropic |
| HGNC:100 | ASIC1 | acid sensing ion channel subunit 1 | Approved | gene with protein product | ACCN2 | BNaC2, hBNaC2 | 12q13.12 | 41 | ENSG00000110881 | OTTHUMG00000169812 | 290 | Acid sensing ion channel subunits |
| HGNC:99 | ASIC2 | acid sensing ion channel subunit 2 | Approved | gene with protein product | ACCN, ACCN1 | ASIC2a, BNC1, BNaC1, hBNaC1, MDEG | 17q11.2-q12 | 40 | ENSG00000108684 | OTTHUMG00000132885 | 290 | Acid sensing ion channel subunits |
| HGNC:101 | ASIC3 | acid sensing ion channel subunit 3 | Approved | gene with protein product | ACCN3 | TNaC1, DRASIC | 7q36.1 | 9311 | ENSG00000213199 | OTTHUMG00000158685 | 290 | Acid sensing ion channel subunits |
| HGNC:21263 | ASIC4 | acid sensing ion channel subunit family member 4 | Approved | gene with protein product | ACCN4 | BNAC4 | 2q35 | 55515 | ENSG00000072182 | OTTHUMG00000058928 | 290 | Acid sensing ion channel subunits |
| HGNC:17537 | ASIC5 | acid sensing ion channel subunit family member 5 | Approved | gene with protein product | ACCN5 | INAC, HINAC | 4q32.1 | 51802 | ENSG00000256394 | OTTHUMG00000161941 | 290 | Acid sensing ion channel subunits |
| HGNC:21625 | ANO1 | anoctamin 1 | Approved | gene with protein product | ORAOV2, TMEM16A | TAOS2, FLJ10261, DOG1 | 11q13.3 | 55107 | ENSG00000131620 | OTTHUMG00000167204 | 865 | Anoctamins |
| HGNC:25519 | ANO10 | anoctamin 10 | Approved | gene with protein product | TMEM16K | FLJ10375, MGC47890, SCAR10 | 3p22.1-p21.33 | 55129 | ENSG00000160746 | OTTHUMG00000133044 | 865 | Anoctamins |
| HGNC:1183 | ANO2 | anoctamin 2 | Approved | gene with protein product | C12orf3, TMEM16B | | 12p13.31 | 57101 | ENSG00000047617 | OTTHUMG00000168264 | 865 | Anoctamins |
| HGNC:14004 | ANO3 | anoctamin 3 | Approved | gene with protein product | C11orf25, TMEM16C | GENX-3947, DYT23 | 11p14.2 | 63982 | ENSG00000134343 | OTTHUMG00000166096 | 865 | Anoctamins |
| HGNC:23837 | ANO4 | anoctamin 4 | Approved | gene with protein product | TMEM16D | FLJ34221, FLJ34272, FLJ35277 | 12q23.1 | 121601 | ENSG00000151572 | OTTHUMG00000170465 | 865 | Anoctamins |
| HGNC:27337 | ANO5 | anoctamin 5 | Approved | gene with protein product | TMEM16E, LGMD2L | GDD1 | 11p14.3 | 203859 | ENSG00000171714 | OTTHUMG00000166051 | 865 | Anoctamins |
| HGNC:25240 | ANO6 | anoctamin 6 | Approved | gene with protein product | TMEM16F | DKFZp313M0720 | 12q12 | 196527 | ENSG00000177119 | OTTHUMG00000169564 | 865 | Anoctamins |
| HGNC:31677 | ANO7 | anoctamin 7 | Approved | gene with protein product | PCANAP5, TMEM16G | NGEP, PCANAP5L, IPCA-5 | 2q37.3 | 50636 | ENSG00000146205 | OTTHUMG00000151702 | 865 | Anoctamins |
| HGNC:29329 | ANO8 | anoctamin 8 | Approved | gene with protein product | KIAA1623, TMEM16H | | 19p13.11 | 57719 | ENSG00000074855 | OTTHUMG00000182665 | 865 | Anoctamins |
| HGNC:20679 | ANO9 | anoctamin 9 | Approved | gene with protein product | TP53I5, TMEM16J | PIG5 | 11p15.5 | 338440 | ENSG00000185101 | OTTHUMG00000165446 | 865 | Anoctamins |
| HGNC:7103 | MIP | major intrinsic protein of lens fiber | Approved | gene with protein product | | MP26, LIM1, AQP0 | 12q13.3 | 4284 | ENSG00000135517 | OTTHUMG00000170571 | 305 | Aquaporins |
| HGNC:633 | AQP1 | aquaporin 1 (Colton blood group) | Approved | gene with protein product | CO | CHIP28 | 7p14.3 | 358 | ENSG00000240583 | OTTHUMG00000023944 | 305 | Aquaporins |
| HGNC:16029 | AQP10 | aquaporin 10 | Approved | gene with protein product | | | 1q21.3 | 89872 | ENSG00000143595 | OTTHUMG00000035980 | 305 | Aquaporins |
| HGNC:19940 | AQP11 | aquaporin 11 | Approved | gene with protein product | | AQPX1 | 11q14.1 | 282679 | ENSG00000178301 | OTTHUMG00000165194 | 305 | Aquaporins |
| HGNC:19941 | AQP12A | aquaporin 12A | Approved | gene with protein product | AQP12 |  | 2q37.3 | 375318 | ENSG00000184945 | OTTHUMG00000183906 | 305 | Aquaporins |
| HGNC:6096 | AQP12B | aquaporin 12B | Approved | gene with protein product | INSSA3 |  | 2q37.3 | 653437 | ENSG00000185176 | OTTHUMG00000152263 | 305 | Aquaporins |
| HGNC:634 | AQP2 | aquaporin 2 | Approved | gene with protein product | | | 12q13.12 | 359 | ENSG00000167580 | OTTHUMG00000169709 | 305 | Aquaporins |
| HGNC:636 | AQP3 | aquaporin 3 (Gill blood group) | Approved | gene with protein product | | GIL | 9p13.3 | 360 | ENSG00000165272 | OTTHUMG00000019769 | 305 | Aquaporins |
| HGNC:637 | AQP4 | aquaporin 4 | Approved | gene with protein product | | MIWC, AQP-4, hAQP4 | 18q11.2 | 361 | ENSG00000171885 | OTTHUMG00000131955 | 305 | Aquaporins |
| HGNC:638 | AQP5 | aquaporin 5 | Approved | gene with protein product | | | 12q13.12 | 362 | ENSG00000161798 | OTTHUMG00000169710 | 305 | Aquaporins |
| HGNC:639 | AQP6 | aquaporin 6 | Approved | gene with protein product | AQP2L |  | 12q13.12 | 363 | ENSG00000086159 | OTTHUMG00000133548 | 305 | Aquaporins |
| HGNC:640 | AQP7 | aquaporin 7 | Approved | gene with protein product | AQP7L | AQP9, AQPap | 9p13.3 | 364 | ENSG00000165269 | OTTHUMG00000019773 | 305 | Aquaporins |
| HGNC:642 | AQP8 | aquaporin 8 | Approved | gene with protein product | | | 16p12.1 | 343 | ENSG00000103375 | OTTHUMG00000097013 | 305 | Aquaporins |
| HGNC:643 | AQP9 | aquaporin 9 | Approved | gene with protein product | | SSC1, HsT17287 | 15q21.3 | 366 | ENSG00000103569 | OTTHUMG00000132631 | 305 | Aquaporins |
| HGNC:12703 | BEST1 | bestrophin 1 | Approved | gene with protein product | VMD2 | BMD, BEST, RP50 | 11q12.3 | 7439 | ENSG00000167995 | OTTHUMG00000167469 | 866 | Bestrophins |
| HGNC:17107 | BEST2 | bestrophin 2 | Approved | gene with protein product | VMD2L1 | FLJ20132 | 19p13.13 | 54831 | ENSG00000039987 | OTTHUMG00000169293 | 866 | Bestrophins |
| HGNC:17105 | BEST3 | bestrophin 3 | Approved | gene with protein product | VMD2L3 | MGC40411, MGC13168 | 12q15 | 144453 | ENSG00000127325 | OTTHUMG00000149919 | 866 | Bestrophins |
| HGNC:17106 | BEST4 | bestrophin 4 | Approved | gene with protein product | VMD2L2 |  | 1p34.1 | 266675 | ENSG00000142959 | OTTHUMG00000008488 | 866 | Bestrophins |
| HGNC:1405 | CACNG1 | calcium voltage-gated channel auxiliary subunit gamma 1 | Approved | gene with protein product | CACNLG |  | 17q24.2 | 786 | ENSG00000108878 | OTTHUMG00000179553 | 1511 | Calcium channel auxiliary gamma subunits |
| HGNC:1406 | CACNG2 | calcium voltage-gated channel auxiliary subunit gamma 2 | Approved | gene with protein product | | stargazin, MGC138502, MGC138504 | 22q12.3 | 10369 | ENSG00000166862 | OTTHUMG00000030612 | 1511 | Calcium channel auxiliary gamma subunits |
| HGNC:1407 | CACNG3 | calcium voltage-gated channel auxiliary subunit gamma 3 | Approved | gene with protein product | | | 16p12.1 | 10368 | ENSG00000006116 | OTTHUMG00000131651 | 1511 | Calcium channel auxiliary gamma subunits |
| HGNC:1408 | CACNG4 | calcium voltage-gated channel auxiliary subunit gamma 4 | Approved | gene with protein product | | MGC11138, MGC24983 | 17q24.2 | 27092 | ENSG00000075461 | OTTHUMG00000179550 | 1511 | Calcium channel auxiliary gamma subunits |
| HGNC:1409 | CACNG5 | calcium voltage-gated channel auxiliary subunit gamma 5 | Approved | gene with protein product | | | 17q24.2 | 27091 | ENSG00000075429 | OTTHUMG00000166468 | 1511 | Calcium channel auxiliary gamma subunits |
| HGNC:13625 | CACNG6 | calcium voltage-gated channel auxiliary subunit gamma 6 | Approved | gene with protein product | | | 19q13.42 | 59285 | ENSG00000130433 | OTTHUMG00000064907 | 1511 | Calcium channel auxiliary gamma subunits |
| HGNC:13626 | CACNG7 | calcium voltage-gated channel auxiliary subunit gamma 7 | Approved | gene with protein product | | | 19q13.42 | 59284 | ENSG00000105605 | OTTHUMG00000064852 | 1511 | Calcium channel auxiliary gamma subunits |
| HGNC:13628 | CACNG8 | calcium voltage-gated channel auxiliary subunit gamma 8 | Approved | gene with protein product | | | 19q13.42 | 59283 | ENSG00000142408 | OTTHUMG00000064908 | 1511 | Calcium channel auxiliary gamma subunits |
| HGNC:1388 | CACNA1A | calcium voltage-gated channel subunit alpha1 A | Approved | gene with protein product | CACNL1A4, SCA6, MHP1, MHP | Cav2.1, EA2, APCA, HPCA, FHM | 19p13.13 | 773 | ENSG00000141837 | OTTHUMG00000044590 | 1512 | Calcium voltage-gated channel alpha1 subunits |
| HGNC:1389 | CACNA1B | calcium voltage-gated channel subunit alpha1 B | Approved | gene with protein product | CACNL1A5 | Cav2.2, CACNN | 9q34.3 | 774 | ENSG00000148408 | OTTHUMG00000021002 | 1512 | Calcium voltage-gated channel alpha1 subunits |
| HGNC:1390 | CACNA1C | calcium voltage-gated channel subunit alpha1 C | Approved | gene with protein product | CCHL1A1, CACNL1A1, CACNA1C-IT2 | Cav1.2, CACH2, CACN2, TS, LQT8 | 12p13.33 | 775 | ENSG00000151067 | OTTHUMG00000150243 | 1512 | Calcium voltage-gated channel alpha1 subunits |
| HGNC:1391 | CACNA1D | calcium voltage-gated channel subunit alpha1 D | Approved | gene with protein product | CCHL1A2, CACNL1A2 | Cav1.3, CACH3, CACN4 | 3p21.1 | 776 | ENSG00000157388 | OTTHUMG00000158278 | 1512 | Calcium voltage-gated channel alpha1 subunits |
| HGNC:1392 | CACNA1E | calcium voltage-gated channel subunit alpha1 E | Approved | gene with protein product | CACNL1A6 | Cav2.3, BII, CACH6 | 1q25.3 | 777 | ENSG00000198216 | OTTHUMG00000037301 | 1512 | Calcium voltage-gated channel alpha1 subunits |
| HGNC:1393 | CACNA1F | calcium voltage-gated channel subunit alpha1 F | Approved | gene with protein product | CSNB2, AIED | Cav1.4, JM8, JMC8, CSNBX2, CORDX3, CSNB2A, OA2 | Xp11.23 | 778 | ENSG00000102001 | OTTHUMG00000022703 | 1512 | Calcium voltage-gated channel alpha1 subunits |
| HGNC:1394 | CACNA1G | calcium voltage-gated channel subunit alpha1 G | Approved | gene with protein product | | Cav3.1, NBR13 | 17q21.33 | 8913 | ENSG00000006283 | OTTHUMG00000162180 | 1512 | Calcium voltage-gated channel alpha1 subunits |
| HGNC:1395 | CACNA1H | calcium voltage-gated channel subunit alpha1 H | Approved | gene with protein product | | Cav3.2 | 16p13.3 | 8912 | ENSG00000196557 | OTTHUMG00000172992 | 1512 | Calcium voltage-gated channel alpha1 subunits |
| HGNC:1396 | CACNA1I | calcium voltage-gated channel subunit alpha1 I | Approved | gene with protein product | | Cav3.3 | 22q13.1 | 8911 | ENSG00000100346 | OTTHUMG00000151096 | 1512 | Calcium voltage-gated channel alpha1 subunits |
| HGNC:1397 | CACNA1S | calcium voltage-gated channel subunit alpha1 S | Approved | gene with protein product | HOKPP, MHS5, CACNL1A3 | Cav1.1, hypoPP | 1q32.1 | 779 | ENSG00000081248 | OTTHUMG00000035784 | 1512 | Calcium voltage-gated channel alpha1 subunits |
| HGNC:1399 | CACNA2D1 | calcium voltage-gated channel auxiliary subunit alpha2delta 1 | Approved | gene with protein product | CACNL2A, CACNA2, MHS3, LINC01112 | lncRNA-N3, alpha2delta-1 | 7q21.11 | 781 | ENSG00000153956 | OTTHUMG00000023622 | 1514 | Calcium voltage-gated channel auxiliary alpha2delta subunits |
| HGNC:1400 | CACNA2D2 | calcium voltage-gated channel auxiliary subunit alpha2delta 2 | Approved | gene with protein product | | KIAA0558, alpha2delta-2 | 3p21.31 | 9254 | ENSG00000007402 | OTTHUMG00000156887 | 1514 | Calcium voltage-gated channel auxiliary alpha2delta subunits |
| HGNC:15460 | CACNA2D3 | calcium voltage-gated channel auxiliary subunit alpha2delta 3 | Approved | gene with protein product | | HSA272268, alpha2delta-3 | 3p21.1-p14.3 | 55799 | ENSG00000157445 | OTTHUMG00000158580 | 1514 | Calcium voltage-gated channel auxiliary alpha2delta subunits |
| HGNC:20202 | CACNA2D4 | calcium voltage-gated channel auxiliary subunit alpha2delta 4 | Approved | gene with protein product | | alpha2delta-4 | 12p13.33 | 93589 | ENSG00000151062 | OTTHUMG00000168111 | 1514 | Calcium voltage-gated channel auxiliary alpha2delta subunits |
| HGNC:1401 | CACNB1 | calcium voltage-gated channel auxiliary subunit beta 1 | Approved | gene with protein product | CACNLB1 |  | 17q12 | 782 | ENSG00000067191 | OTTHUMG00000133217 | 1515 | Calcium voltage-gated channel auxiliary beta subunits |
| HGNC:1402 | CACNB2 | calcium voltage-gated channel auxiliary subunit beta 2 | Approved | gene with protein product | MYSB, CACNLB2 | | 10p12 | 783 | ENSG00000165995 | OTTHUMG00000017764 | 1515 | Calcium voltage-gated channel auxiliary beta subunits |
| HGNC:1403 | CACNB3 | calcium voltage-gated channel auxiliary subunit beta 3 | Approved | gene with protein product | CACNLB3 |  | 12q13.12 | 784 | ENSG00000167535 | OTTHUMG00000170398 | 1515 | Calcium voltage-gated channel auxiliary beta subunits |
| HGNC:1404 | CACNB4 | calcium voltage-gated channel auxiliary subunit beta 4 | Approved | gene with protein product | | EJM4 | 2q23.3 | 785 | ENSG00000182389 | OTTHUMG00000155091 | 1515 | Calcium voltage-gated channel auxiliary beta subunits |
| HGNC:17116 | CATSPER1 | cation channel sperm associated 1 | Approved | gene with protein product | | CATSPER | 11q13.1 | 117144 | ENSG00000175294 | OTTHUMG00000166668 | 186 | Cation channels sperm associated |
| HGNC:18810 | CATSPER2 | cation channel sperm associated 2 | Approved | gene with protein product | | | 15q15.3 | 117155 | ENSG00000166762 | OTTHUMG00000059902 | 186 | Cation channels sperm associated |
| HGNC:20819 | CATSPER3 | cation channel sperm associated 3 | Approved | gene with protein product | | CACRC | 5q31.1 | 347732 | ENSG00000152705 | OTTHUMG00000129137 | 186 | Cation channels sperm associated |
| HGNC:23220 | CATSPER4 | cation channel sperm associated 4 | Approved | gene with protein product | | | 1p36.11 | 378807 | ENSG00000188782 | OTTHUMG00000003383 | 186 | Cation channels sperm associated |
| HGNC:20500 | CATSPERB | cation channel sperm associated auxiliary subunit beta | Approved | gene with protein product | C14orf161 | FLJ14298 | 14q32.12 | 79820 | ENSG00000133962 | OTTHUMG00000171118 | 186 | Cation channels sperm associated |
| HGNC:28598 | CATSPERD | cation channel sperm associated auxiliary subunit delta | Approved | gene with protein product | TMEM146 | MGC39581 | 19p13.3 | 257062 | ENSG00000174898 | OTTHUMG00000143036 | 186 | Cation channels sperm associated |
| HGNC:28491 | CATSPERE | catsper channel auxiliary subunit epsilon | Approved | gene with protein product | C1orf101 | MGC33370 | 1q44 | 257044 | ENSG00000179397 | OTTHUMG00000040103 | 186 | Cation channels sperm associated |
| HGNC:25243 | CATSPERG | cation channel sperm associated auxiliary subunit gamma | Approved | gene with protein product | C19orf15 | DKFZp434A1022, FLJ46353 | 19q13.2 | 57828 | ENSG00000099338 | OTTHUMG00000153223 | 186 | Cation channels sperm associated |
| HGNC:19231 | CATSPERZ | catsper channel auxiliary subunit zeta | Approved | gene with protein product | C11orf20, TEX40 | DKFZP566E164 | 11q13.1 | 25858 | ENSG00000219435 | OTTHUMG00000167820 | 186 | Cation channels sperm associated |
| HGNC:1884 | CFTR | CF transmembrane conductance regulator | Approved | gene with protein product | CF, ABCC7 | MRP7, ABC35, TNR-CFTR, dJ760C5.1, CFTR/MRP | 7q31.2 | 1080 | ENSG00000001626 | OTTHUMG00000023076 | 309 | Chloride channels, ATP-gated CFTR |
| HGNC:2062 | CLIC1 | chloride intracellular channel 1 | Approved | gene with protein product | | NCC27, p64CLCP, G6, CLCNL1 | 6p21.33 | 1192 | ENSG00000213719 | OTTHUMG00000031103 | 307 | Chloride intracellular channels |
| HGNC:2063 | CLIC2 | chloride intracellular channel 2 | Approved | gene with protein product | | XAP121, CLCNL2 | Xq28 | 1193 | ENSG00000155962 | OTTHUMG00000022660 | 307 | Chloride intracellular channels |
| HGNC:2064 | CLIC3 | chloride intracellular channel 3 | Approved | gene with protein product | | | 9q34.3 | 9022 | ENSG00000169583 | OTTHUMG00000020954 | 307 | Chloride intracellular channels |
| HGNC:13518 | CLIC4 | chloride intracellular channel 4 | Approved | gene with protein product | | DKFZP566G223, CLIC4L, P64H1, H1, huH1, p64H1 | 1p36.11 | 25932 | ENSG00000169504 | OTTHUMG00000003327 | 307 | Chloride intracellular channels |
| HGNC:13517 | CLIC5 | chloride intracellular channel 5 | Approved | gene with protein product | | DFNB102 | 6p21.1 | 53405 | ENSG00000112782 | OTTHUMG00000014775 | 307 | Chloride intracellular channels |
| HGNC:2065 | CLIC6 | chloride intracellular channel 6 | Approved | gene with protein product | CLIC1L | CLIC5 | 21q22.12 | 54102 | ENSG00000159212 | OTTHUMG00000086237 | 307 | Chloride intracellular channels |
| HGNC:2019 | CLCN1 | chloride voltage-gated channel 1 | Approved | gene with protein product | | CLC1, ClC-1 | 7q34 | 1180 | ENSG00000188037 | OTTHUMG00000152695 | 302 | Chloride voltage-gated channels |
| HGNC:2020 | CLCN2 | chloride voltage-gated channel 2 | Approved | gene with protein product | | CLC2, EJM6, ClC-2 | 3q27.1 | 1181 | ENSG00000114859 | OTTHUMG00000156747 | 302 | Chloride voltage-gated channels |
| HGNC:2021 | CLCN3 | chloride voltage-gated channel 3 | Approved | gene with protein product | | CLC3, ClC-3 | 4q33 | 1182 | ENSG00000109572 | OTTHUMG00000160973 | 302 | Chloride voltage-gated channels |
| HGNC:2022 | CLCN4 | chloride voltage-gated channel 4 | Approved | gene with protein product | | CLC4, ClC-4 | Xp22.2 | 1183 | ENSG00000073464 | OTTHUMG00000021125 | 302 | Chloride voltage-gated channels |
| HGNC:2023 | CLCN5 | chloride voltage-gated channel 5 | Approved | gene with protein product | NPHL2, NPHL1 | DENTS, XLRH, hClC-K2, hCIC-K2, CLC5, XRN, ClC-5 | Xp11.23 | 1184 | ENSG00000171365 | OTTHUMG00000021514 | 302 | Chloride voltage-gated channels |
| HGNC:2024 | CLCN6 | chloride voltage-gated channel 6 | Approved | gene with protein product | | CLC-6, KIAA0046, ClC-6 | 1p36.22 | 1185 | ENSG00000011021 | OTTHUMG00000002299 | 302 | Chloride voltage-gated channels |
| HGNC:2025 | CLCN7 | chloride voltage-gated channel 7 | Approved | gene with protein product | | CLC-7, OPTA2, CLC7, ClC-7, PPP1R63 | 16p13.3 | 1186 | ENSG00000103249 | OTTHUMG00000044467 | 302 | Chloride voltage-gated channels |
| HGNC:2026 | CLCNKA | chloride voltage-gated channel Ka | Approved | gene with protein product | | hClC-Ka, ClC-K1, CLCK1 | 1p36.13 | 1187 | ENSG00000186510 | OTTHUMG00000009529 | 302 | Chloride voltage-gated channels |
| HGNC:2027 | CLCNKB | chloride voltage-gated channel Kb | Approved | gene with protein product | | hClC-Kb, CLCKB, ClC-K2, ClC-Kb | 1p36.13 | 1188 | ENSG00000184908 | OTTHUMG00000009530 | 302 | Chloride voltage-gated channels |
| HGNC:16512 | BSND | barttin CLCNK type accessory subunit beta | Approved | gene with protein product | DFNB73 | BART | 1p32.3 | 7809 | ENSG00000162399 | OTTHUMG00000008112 | 302 | Chloride voltage-gated channels |
| HGNC:1955 | CHRNA1 | cholinergic receptor nicotinic alpha 1 subunit | Approved | gene with protein product | CHRNA |  | 2q31.1 | 1134 | ENSG00000138435 | OTTHUMG00000132357 | 173 | Cholinergic receptors nicotinic subunits |
| HGNC:13800 | CHRNA10 | cholinergic receptor nicotinic alpha 10 subunit | Approved | gene with protein product | | | 11p15.4 | 57053 | ENSG00000129749 | OTTHUMG00000011844 | 173 | Cholinergic receptors nicotinic subunits |
| HGNC:1956 | CHRNA2 | cholinergic receptor nicotinic alpha 2 subunit | Approved | gene with protein product | | | 8p21.2 | 1135 | ENSG00000120903 | OTTHUMG00000102083 | 173 | Cholinergic receptors nicotinic subunits |
| HGNC:1957 | CHRNA3 | cholinergic receptor nicotinic alpha 3 subunit | Approved | gene with protein product | | | 15q25.1 | 1136 | ENSG00000080644 | OTTHUMG00000143863 | 173 | Cholinergic receptors nicotinic subunits |
| HGNC:1958 | CHRNA4 | cholinergic receptor nicotinic alpha 4 subunit | Approved | gene with protein product | EBN, EBN1 | BFNC | 20q13.33 | 1137 | ENSG00000101204 | OTTHUMG00000033080 | 173 | Cholinergic receptors nicotinic subunits |
| HGNC:1959 | CHRNA5 | cholinergic receptor nicotinic alpha 5 subunit | Approved | gene with protein product | | | 15q25.1 | 1138 | ENSG00000169684 | OTTHUMG00000143858 | 173 | Cholinergic receptors nicotinic subunits |
| HGNC:15963 | CHRNA6 | cholinergic receptor nicotinic alpha 6 subunit | Approved | gene with protein product | | | 8p11.21 | 8973 | ENSG00000147434 | OTTHUMG00000165275 | 173 | Cholinergic receptors nicotinic subunits |
| HGNC:1960 | CHRNA7 | cholinergic receptor nicotinic alpha 7 subunit | Approved | gene with protein product | | | 15q13.3 | 1139 | ENSG00000175344 | OTTHUMG00000129285 | 173 | Cholinergic receptors nicotinic subunits |
| HGNC:14079 | CHRNA9 | cholinergic receptor nicotinic alpha 9 subunit | Approved | gene with protein product | | NACHRA9 | 4p14 | 55584 | ENSG00000174343 | OTTHUMG00000099375 | 173 | Cholinergic receptors nicotinic subunits |
| HGNC:1961 | CHRNB1 | cholinergic receptor nicotinic beta 1 subunit | Approved | gene with protein product | CHRNB |  | 17p13.1 | 1140 | ENSG00000170175 | OTTHUMG00000108139 | 173 | Cholinergic receptors nicotinic subunits |
| HGNC:1962 | CHRNB2 | cholinergic receptor nicotinic beta 2 subunit | Approved | gene with protein product | | | 1q21.3 | 1141 | ENSG00000160716 | OTTHUMG00000037262 | 173 | Cholinergic receptors nicotinic subunits |
| HGNC:1963 | CHRNB3 | cholinergic receptor nicotinic beta 3 subunit | Approved | gene with protein product | | | 8p11.21 | 1142 | ENSG00000147432 | OTTHUMG00000165262 | 173 | Cholinergic receptors nicotinic subunits |
| HGNC:1964 | CHRNB4 | cholinergic receptor nicotinic beta 4 subunit | Approved | gene with protein product | | | 15q25.1 | 1143 | ENSG00000117971 | OTTHUMG00000143860 | 173 | Cholinergic receptors nicotinic subunits |
| HGNC:1965 | CHRND | cholinergic receptor nicotinic delta subunit | Approved | gene with protein product | ACHRD |  | 2q37.1 | 1144 | ENSG00000135902 | OTTHUMG00000133261 | 173 | Cholinergic receptors nicotinic subunits |
| HGNC:1966 | CHRNE | cholinergic receptor nicotinic epsilon subunit | Approved | gene with protein product | | ACHRE | 17p13.2 | 1145 | ENSG00000108556 | OTTHUMG00000090778 | 173 | Cholinergic receptors nicotinic subunits |
| HGNC:1967 | CHRNG | cholinergic receptor nicotinic gamma subunit | Approved | gene with protein product | ACHRG |  | 2q37.1 | 1146 | ENSG00000196811 | OTTHUMG00000153327 | 173 | Cholinergic receptors nicotinic subunits |
| HGNC:2148 | CNGA1 | cyclic nucleotide gated channel subunit alpha 1 | Approved | gene with protein product | CNCG1, CNCG | RCNC1, RCNCa, CNG1, RP49 | 4p12 | 1259 | ENSG00000198515 | OTTHUMG00000160668 | 250 | Cyclic nucleotide gated channels |
| HGNC:2149 | CNGA2 | cyclic nucleotide gated channel subunit alpha 2 | Approved | gene with protein product | CNCA1, CNCA | CNG2, OCNC1, OCNCa, OCNCALPHA, OCNCalpha, FLJ46312 | Xq28 | 1260 | ENSG00000183862 | OTTHUMG00000024173 | 250 | Cyclic nucleotide gated channels |
| HGNC:2150 | CNGA3 | cyclic nucleotide gated channel subunit alpha 3 | Approved | gene with protein product | CNCG3, ACHM2 | CCNC1, CCNCa, CNG3 | 2q11.2 | 1261 | ENSG00000144191 | OTTHUMG00000130561 | 250 | Cyclic nucleotide gated channels |
| HGNC:2152 | CNGA4 | cyclic nucleotide gated channel subunit alpha 4 | Approved | gene with protein product | CNCA2, CNGB2 | OCNC2, OCNCb, CNG5 | 11p15.4 | 1262 | ENSG00000132259 | OTTHUMG00000165379 | 250 | Cyclic nucleotide gated channels |
| HGNC:2151 | CNGB1 | cyclic nucleotide gated channel subunit beta 1 | Approved | gene with protein product | CNCG2, CNCG3L | RCNC2, RCNCb, GARP, GAR1, CNGB1B, RP45 | 16q21 | 1258 | ENSG00000070729 | OTTHUMG00000154810 | 250 | Cyclic nucleotide gated channels |
| HGNC:2153 | CNGB3 | cyclic nucleotide gated channel subunit beta 3 | Approved | gene with protein product | ACHM3, ACHM1, RMCH | | 8q21.3 | 54714 | ENSG00000170289 | OTTHUMG00000163738 | 250 | Cyclic nucleotide gated channels |
| HGNC:4845 | HCN1 | hyperpolarization activated cyclic nucleotide gated potassium channel 1 | Approved | gene with protein product | BCNG1 | BCNG-1, HAC-2 | 5p12 | 348980 | ENSG00000164588 | OTTHUMG00000131155 | 250 | Cyclic nucleotide gated channels |
| HGNC:4846 | HCN2 | hyperpolarization activated cyclic nucleotide gated potassium and sodium channel 2 | Approved | gene with protein product | BCNG2 | BCNG-2, HAC-1 | 19p13.3 | 610 | ENSG00000099822 | OTTHUMG00000180590 | 250 | Cyclic nucleotide gated channels |
| HGNC:19183 | HCN3 | hyperpolarization activated cyclic nucleotide gated potassium channel 3 | Approved | gene with protein product | | KIAA1535 | 1q22 | 57657 | ENSG00000143630 | OTTHUMG00000035872 | 250 | Cyclic nucleotide gated channels |
| HGNC:16882 | HCN4 | hyperpolarization activated cyclic nucleotide gated potassium channel 4 | Approved | gene with protein product | | | 15q24.1 | 10021 | ENSG00000138622 | OTTHUMG00000137563 | 250 | Cyclic nucleotide gated channels |
| HGNC:4075 | GABRA1 | gamma-aminobutyric acid type A receptor subunit alpha1 | Approved | gene with protein product | | EJM5 | 5q34 | 2554 | ENSG00000022355 | OTTHUMG00000163586 | 563 | Gamma-aminobutyric acid type A receptor subunits |
| HGNC:4076 | GABRA2 | gamma-aminobutyric acid type A receptor subunit alpha2 | Approved | gene with protein product | | | 4p12 | 2555 | ENSG00000151834 | OTTHUMG00000044266 | 563 | Gamma-aminobutyric acid type A receptor subunits |
| HGNC:4077 | GABRA3 | gamma-aminobutyric acid type A receptor subunit alpha3 | Approved | gene with protein product | | | Xq28 | 2556 | ENSG00000011677 | OTTHUMG00000024183 | 563 | Gamma-aminobutyric acid type A receptor subunits |
| HGNC:4078 | GABRA4 | gamma-aminobutyric acid type A receptor subunit alpha4 | Approved | gene with protein product | | | 4p12 | 2557 | ENSG00000109158 | OTTHUMG00000099431 | 563 | Gamma-aminobutyric acid type A receptor subunits |
| HGNC:4079 | GABRA5 | gamma-aminobutyric acid type A receptor subunit alpha5 | Approved | gene with protein product | | | 15q12 | 2558 | ENSG00000186297 | OTTHUMG00000171824 | 563 | Gamma-aminobutyric acid type A receptor subunits |
| HGNC:4080 | GABRA6 | gamma-aminobutyric acid type A receptor subunit alpha6 | Approved | gene with protein product | | | 5q34 | 2559 | ENSG00000145863 | OTTHUMG00000130351 | 563 | Gamma-aminobutyric acid type A receptor subunits |
| HGNC:4081 | GABRB1 | gamma-aminobutyric acid type A receptor subunit beta1 | Approved | gene with protein product | | | 4p12 | 2560 | ENSG00000163288 | OTTHUMG00000044269 | 563 | Gamma-aminobutyric acid type A receptor subunits |
| HGNC:4082 | GABRB2 | gamma-aminobutyric acid type A receptor subunit beta2 | Approved | gene with protein product | | | 5q34 | 2561 | ENSG00000145864 | OTTHUMG00000130349 | 563 | Gamma-aminobutyric acid type A receptor subunits |
| HGNC:4083 | GABRB3 | gamma-aminobutyric acid type A receptor subunit beta3 | Approved | gene with protein product | | | 15q12 | 2562 | ENSG00000166206 | OTTHUMG00000129231 | 563 | Gamma-aminobutyric acid type A receptor subunits |
| HGNC:4084 | GABRD | gamma-aminobutyric acid type A receptor subunit delta | Approved | gene with protein product | | GABAARdelta | 1p36.33 | 2563 | ENSG00000187730 | OTTHUMG00000041064 | 563 | Gamma-aminobutyric acid type A receptor subunits |
| HGNC:4085 | GABRE | gamma-aminobutyric acid type A receptor subunit epsilon | Approved | gene with protein product | | | Xq28 | 2564 | ENSG00000102287 | OTTHUMG00000024176 | 563 | Gamma-aminobutyric acid type A receptor subunits |
| HGNC:4086 | GABRG1 | gamma-aminobutyric acid type A receptor subunit gamma1 | Approved | gene with protein product | | | 4p12 | 2565 | ENSG00000163285 | OTTHUMG00000128609 | 563 | Gamma-aminobutyric acid type A receptor subunits |
| HGNC:4087 | GABRG2 | gamma-aminobutyric acid type A receptor subunit gamma2 | Approved | gene with protein product | | | 5q34 | 2566 | ENSG00000113327 | OTTHUMG00000130350 | 563 | Gamma-aminobutyric acid type A receptor subunits |
| HGNC:4088 | GABRG3 | gamma-aminobutyric acid type A receptor subunit gamma3 | Approved | gene with protein product | | | 15q12 | 2567 | ENSG00000182256 | OTTHUMG00000044462 | 563 | Gamma-aminobutyric acid type A receptor subunits |
| HGNC:4089 | GABRP | gamma-aminobutyric acid type A receptor subunit pi | Approved | gene with protein product | | | 5q35.1 | 2568 | ENSG00000094755 | OTTHUMG00000130443 | 563 | Gamma-aminobutyric acid type A receptor subunits |
| HGNC:14454 | GABRQ | gamma-aminobutyric acid type A receptor subunit theta | Approved | gene with protein product | | | Xq28 | 55879 | ENSG00000268089 | OTTHUMG00000022649 | 563 | Gamma-aminobutyric acid type A receptor subunits |
| HGNC:4090 | GABRR1 | gamma-aminobutyric acid type A receptor subunit rho1 | Approved | gene with protein product | | | 6q15 | 2569 | ENSG00000146276 | OTTHUMG00000015195 | 563 | Gamma-aminobutyric acid type A receptor subunits |
| HGNC:4091 | GABRR2 | gamma-aminobutyric acid type A receptor subunit rho2 | Approved | gene with protein product | | | 6q15 | 2570 | ENSG00000111886 | OTTHUMG00000015198 | 563 | Gamma-aminobutyric acid type A receptor subunits |
| HGNC:17969 | GABRR3 | gamma-aminobutyric acid type A receptor subunit rho3 | Approved | gene with protein product | | | 3q11.2 | 200959 | ENSG00000183185 | OTTHUMG00000159135 | 563 | Gamma-aminobutyric acid type A receptor subunits |
| HGNC:4274 | GJA1 | gap junction protein alpha 1 | Approved | gene with protein product | ODDD, GJAL | CX43, ODD, ODOD, SDTY3 | 6q22.31 | 2697 | ENSG00000152661 | OTTHUMG00000015479 | 314 | Gap junction proteins |
| HGNC:16995 | GJA10 | gap junction protein alpha 10 | Approved | gene with protein product | | CX62 | 6q15 | 84694 | ENSG00000135355 | OTTHUMG00000015210 | 314 | Gap junction proteins |
| HGNC:4277 | GJA3 | gap junction protein alpha 3 | Approved | gene with protein product | CZP3 | CX46 | 13q12.11 | 2700 | ENSG00000121743 | OTTHUMG00000016510 | 314 | Gap junction proteins |
| HGNC:4278 | GJA4 | gap junction protein alpha 4 | Approved | gene with protein product | | CX37 | 1p34.3 | 2701 | ENSG00000187513 | OTTHUMG00000004050 | 314 | Gap junction proteins |
| HGNC:4279 | GJA5 | gap junction protein alpha 5 | Approved | gene with protein product | | CX40 | 1q21.2 | 2702 | ENSG00000265107 | OTTHUMG00000014020 | 314 | Gap junction proteins |
| HGNC:32542 | GJA6P | gap junction protein alpha 6, pseudogene | Approved | pseudogene | |  | Xp22.13 | 1E+08 | ENSG00000236187 | OTTHUMG00000021215 | 314 | Gap junction proteins |
| HGNC:4281 | GJA8 | gap junction protein alpha 8 | Approved | gene with protein product | CAE1, CZP1, CAE | CX50 | 1q21.2 | 2703 | ENSG00000121634 | OTTHUMG00000024085 | 314 | Gap junction proteins |
| HGNC:19155 | GJA9 | gap junction protein alpha 9 | Approved | gene with protein product | GJA10 | CX59, CX58 | 1p34.3 | 81025 | ENSG00000131233 | OTTHUMG00000000482 | 314 | Gap junction proteins |
| HGNC:4283 | GJB1 | gap junction protein beta 1 | Approved | gene with protein product | CMTX1, CMTX | CX32 | Xq13.1 | 2705 | ENSG00000169562 | OTTHUMG00000021797 | 314 | Gap junction proteins |
| HGNC:4284 | GJB2 | gap junction protein beta 2 | Approved | gene with protein product | DFNB1, DFNA3 | CX26, NSRD1 | 13q12.11 | 2706 | ENSG00000165474 | OTTHUMG00000016513 | 314 | Gap junction proteins |
| HGNC:4285 | GJB3 | gap junction protein beta 3 | Approved | gene with protein product | DFNA2, EKV | CX31 | 1p34.3 | 2707 | ENSG00000188910 | OTTHUMG00000004051 | 314 | Gap junction proteins |
| HGNC:4286 | GJB4 | gap junction protein beta 4 | Approved | gene with protein product | | CX30.3 | 1p34.3 | 127534 | ENSG00000189433 | OTTHUMG00000004052 | 314 | Gap junction proteins |
| HGNC:4287 | GJB5 | gap junction protein beta 5 | Approved | gene with protein product | | CX31.1 | 1p34.3 | 2709 | ENSG00000189280 | OTTHUMG00000004053 | 314 | Gap junction proteins |
| HGNC:4288 | GJB6 | gap junction protein beta 6 | Approved | gene with protein product | DFNA3, ED2 | EDH, HED, CX30 | 13q12.11 | 10804 | ENSG00000121742 | OTTHUMG00000016515 | 314 | Gap junction proteins |
| HGNC:16690 | GJB7 | gap junction protein beta 7 | Approved | gene with protein product | | CX25, bA136M9.1 | 6q14.3-q15 | 375519 | ENSG00000164411 | OTTHUMG00000015166 | 314 | Gap junction proteins |
| HGNC:4280 | GJC1 | gap junction protein gamma 1 | Approved | gene with protein product | GJA7 | CX45 | 17q21.31 | 10052 | ENSG00000182963 | OTTHUMG00000179861 | 314 | Gap junction proteins |
| HGNC:17494 | GJC2 | gap junction protein gamma 2 | Approved | gene with protein product | GJA12 | CX47, CX46.6, SPG44 | 1q42.13 | 57165 | ENSG00000198835 | OTTHUMG00000039771 | 314 | Gap junction proteins |
| HGNC:17495 | GJC3 | gap junction protein gamma 3 | Approved | gene with protein product | GJE1 | CX30.2, CX29 | 7q22.1 | 349149 | ENSG00000176402 | OTTHUMG00000156649 | 314 | Gap junction proteins |
| HGNC:19154 | GJD2 | gap junction protein delta 2 | Approved | gene with protein product | GJA9 | CX36 | 15q14 | 57369 | ENSG00000159248 | OTTHUMG00000129674 | 314 | Gap junction proteins |
| HGNC:19147 | GJD3 | gap junction protein delta 3 | Approved | gene with protein product | GJC1 | CX31.9, GJA11, Cx30.2 | 17q21.2 | 125111 | ENSG00000183153 | OTTHUMG00000179634 | 314 | Gap junction proteins |
| HGNC:23296 | GJD4 | gap junction protein delta 4 | Approved | gene with protein product | | CX40.1, FLJ90023 | 10p11.21 | 219770 | ENSG00000177291 | OTTHUMG00000017957 | 314 | Gap junction proteins |
| HGNC:33251 | GJE1 | gap junction protein epsilon 1 | Approved | gene with protein product | | CX23 | 6q24.1 | 1E+08 | ENSG00000203733 | OTTHUMG00000015706 | 314 | Gap junction proteins |
| HGNC:4571 | GRIA1 | glutamate ionotropic receptor AMPA type subunit 1 | Approved | gene with protein product | GLUR1 | GluA1, GLURA | 5q33.2 | 2890 | ENSG00000155511 | OTTHUMG00000130148 | 1200 | Glutamate ionotropic receptor AMPA type subunits |
| HGNC:4572 | GRIA2 | glutamate ionotropic receptor AMPA type subunit 2 | Approved | gene with protein product | GLUR2 | GluA2, GLURB, GluR-K2, GluR-B, GluR-2 | 4q32.1 | 2891 | ENSG00000120251 | OTTHUMG00000133836 | 1200 | Glutamate ionotropic receptor AMPA type subunits |
| HGNC:4573 | GRIA3 | glutamate ionotropic receptor AMPA type subunit 3 | Approved | gene with protein product | GLUR3 | GluA3, GLURC, MRX94, GluR-3, GluR-C, GluR-K3, iGluR3 | Xq25 | 2892 | ENSG00000125675 | OTTHUMG00000022685 | 1200 | Glutamate ionotropic receptor AMPA type subunits |
| HGNC:4574 | GRIA4 | glutamate ionotropic receptor AMPA type subunit 4 | Approved | gene with protein product | GLUR4 | GluA4, GLURD, GluR-4, GluR-D, GLUR4C | 11q22.3 | 2893 | ENSG00000152578 | OTTHUMG00000166236 | 1200 | Glutamate ionotropic receptor AMPA type subunits |
| HGNC:4575 | GRID1 | glutamate ionotropic receptor delta type subunit 1 | Approved | gene with protein product | | GluD1, KIAA1220 | 10q23.1-q23.2 | 2894 | ENSG00000182771 | OTTHUMG00000018650 | 1202 | Glutamate ionotropic receptor delta type subunits |
| HGNC:4576 | GRID2 | glutamate ionotropic receptor delta type subunit 2 | Approved | gene with protein product | | GluD2, GluR-delta-2 | 4q22.1-q22.2 | 2895 | ENSG00000152208 | OTTHUMG00000130975 | 1202 | Glutamate ionotropic receptor delta type subunits |
| HGNC:4579 | GRIK1 | glutamate ionotropic receptor kainate type subunit 1 | Approved | gene with protein product | GLUR5 | GluK1 | 21q21.3 | 2897 | ENSG00000171189 | OTTHUMG00000078879 | 1199 | Glutamate ionotropic receptor kainate type subunits |
| HGNC:4580 | GRIK2 | glutamate ionotropic receptor kainate type subunit 2 | Approved | gene with protein product | GLUR6 | GluK2, MRT6, GluR-6, EAA4, GLUK6 | 6q16.3 | 2898 | ENSG00000164418 | OTTHUMG00000016328 | 1199 | Glutamate ionotropic receptor kainate type subunits |
| HGNC:4581 | GRIK3 | glutamate ionotropic receptor kainate type subunit 3 | Approved | gene with protein product | | GluK3, GLUR7 | 1p34.3 | 2899 | ENSG00000163873 | OTTHUMG00000004189 | 1199 | Glutamate ionotropic receptor kainate type subunits |
| HGNC:4582 | GRIK4 | glutamate ionotropic receptor kainate type subunit 4 | Approved | gene with protein product | GRIK | GluK4, KA1 | 11q23.3 | 2900 | ENSG00000149403 | OTTHUMG00000048255 | 1199 | Glutamate ionotropic receptor kainate type subunits |
| HGNC:4583 | GRIK5 | glutamate ionotropic receptor kainate type subunit 5 | Approved | gene with protein product | GRIK2 | GluK5, KA2 | 19q13.2 | 2901 | ENSG00000105737 | OTTHUMG00000044573 | 1199 | Glutamate ionotropic receptor kainate type subunits |
| HGNC:4584 | GRIN1 | glutamate ionotropic receptor NMDA type subunit 1 | Approved | gene with protein product | NMDAR1 | GluN1, NR1 | 9q34.3 | 2902 | ENSG00000176884 | OTTHUMG00000020976 | 1201 | Glutamate ionotropic receptor NMDA type subunits |
| HGNC:4585 | GRIN2A | glutamate ionotropic receptor NMDA type subunit 2A | Approved | gene with protein product | NMDAR2A | GluN2A, NR2A | 16p13.2 | 2903 | ENSG00000183454 | OTTHUMG00000129721 | 1201 | Glutamate ionotropic receptor NMDA type subunits |
| HGNC:4586 | GRIN2B | glutamate ionotropic receptor NMDA type subunit 2B | Approved | gene with protein product | NMDAR2B | GluN2B, NR2B | 12p13.1 | 2904 | ENSG00000273079 | OTTHUMG00000137373 | 1201 | Glutamate ionotropic receptor NMDA type subunits |
| HGNC:4587 | GRIN2C | glutamate ionotropic receptor NMDA type subunit 2C | Approved | gene with protein product | NMDAR2C | GluN2C, NR2C | 17q25.1 | 2905 | ENSG00000161509 | OTTHUMG00000044524 | 1201 | Glutamate ionotropic receptor NMDA type subunits |
| HGNC:4588 | GRIN2D | glutamate ionotropic receptor NMDA type subunit 2D | Approved | gene with protein product | NMDAR2D | GluN2D, EB11, NR2D | 19q13.33 | 2906 | ENSG00000105464 | OTTHUMG00000183304 | 1201 | Glutamate ionotropic receptor NMDA type subunits |
| HGNC:16767 | GRIN3A | glutamate ionotropic receptor NMDA type subunit 3A | Approved | gene with protein product | | GluN3A | 9q31.1 | 116443 | ENSG00000198785 | OTTHUMG00000020387 | 1201 | Glutamate ionotropic receptor NMDA type subunits |
| HGNC:16768 | GRIN3B | glutamate ionotropic receptor NMDA type subunit 3B | Approved | gene with protein product | | GluN3B | 19p13.3 | 116444 | ENSG00000116032 | OTTHUMG00000181904 | 1201 | Glutamate ionotropic receptor NMDA type subunits |
| HGNC:4326 | GLRA1 | glycine receptor alpha 1 | Approved | gene with protein product | STHE |  | 5q33.1 | 2741 | ENSG00000145888 | OTTHUMG00000130121 | 868 | Glycine receptors |
| HGNC:4327 | GLRA2 | glycine receptor alpha 2 | Approved | gene with protein product | GLR |  | Xp22.2 | 2742 | ENSG00000101958 | OTTHUMG00000021166 | 868 | Glycine receptors |
| HGNC:4328 | GLRA3 | glycine receptor alpha 3 | Approved | gene with protein product | | | 4q34.1 | 8001 | ENSG00000145451 | OTTHUMG00000149816 | 868 | Glycine receptors |
| HGNC:31715 | GLRA4 | glycine receptor alpha 4 (pseudogene) | Approved | pseudogene | |  | Xq22.2 | 441509 | ENSG00000188828 | OTTHUMG00000022110 | 868 | Glycine receptors |
| HGNC:4329 | GLRB | glycine receptor beta | Approved | gene with protein product | | | 4q32.1 | 2743 | ENSG00000109738 | OTTHUMG00000161954 | 868 | Glycine receptors |
| HGNC:28240 | HVCN1 | hydrogen voltage gated channel 1 | Approved | gene with protein product | | MGC15619, Hv1, VSOP | 12q24.11 | 84329 | ENSG00000122986 | OTTHUMG00000169530 | 254 | Hydrogen voltage gated channels |
| HGNC:6180 | ITPR1 | inositol 1,4,5-trisphosphate receptor type 1 | Approved | gene with protein product | SCA15, SCA16, SCA29 | Insp3r1, IP3R1, ACV, PPP1R94 | 3p26.1 | 3708 | ENSG00000150995 | OTTHUMG00000154996 | 297 | Inositol 1,4,5-triphosphate receptors |
| HGNC:6181 | ITPR2 | inositol 1,4,5-trisphosphate receptor type 2 | Approved | gene with protein product | | IP3R2, CFAP48 | 12p11.23 | 3709 | ENSG00000123104 | OTTHUMG00000169181 | 297 | Inositol 1,4,5-triphosphate receptors |
| HGNC:6182 | ITPR3 | inositol 1,4,5-trisphosphate receptor type 3 | Approved | gene with protein product | | IP3R3 | 6p21.31 | 3710 | ENSG00000096433 | OTTHUMG00000014532 | 297 | Inositol 1,4,5-triphosphate receptors |
| HGNC:6284 | KCNMA1 | potassium calcium-activated channel subfamily M alpha 1 | Approved | gene with protein product | SLO | KCa1.1, mSLO1 | 10q22.3 | 3778 | ENSG00000156113 | OTTHUMG00000018543 | 255 | Potassium calcium-activated channels |
| HGNC:6290 | KCNN1 | potassium calcium-activated channel subfamily N member 1 | Approved | gene with protein product | | KCa2.1, hSK1 | 19p13.11 | 3780 | ENSG00000105642 | OTTHUMG00000183482 | 255 | Potassium calcium-activated channels |
| HGNC:6291 | KCNN2 | potassium calcium-activated channel subfamily N member 2 | Approved | gene with protein product | | KCa2.2, hSK2 | 5q22.3 | 3781 | ENSG00000080709 | OTTHUMG00000128836 | 255 | Potassium calcium-activated channels |
| HGNC:6292 | KCNN3 | potassium calcium-activated channel subfamily N member 3 | Approved | gene with protein product | | KCa2.3, hSK3, SKCA3 | 1q21.3 | 3782 | ENSG00000143603 | OTTHUMG00000037260 | 255 | Potassium calcium-activated channels |
| HGNC:6293 | KCNN4 | potassium calcium-activated channel subfamily N member 4 | Approved | gene with protein product | | KCa3.1, hSK4, hKCa4, hIKCa1, IK | 19q13.31 | 3783 | ENSG00000104783 | OTTHUMG00000182779 | 255 | Potassium calcium-activated channels |
| HGNC:18867 | KCNU1 | potassium calcium-activated channel subfamily U member 1 | Approved | gene with protein product | | KCa5.1, Slo3, KCNMC1, Kcnma3 | 8p11.23 | 157855 | ENSG00000215262 | OTTHUMG00000163981 | 255 | Potassium calcium-activated channels |
| HGNC:6255 | KCNJ1 | potassium inwardly rectifying channel subfamily J member 1 | Approved | gene with protein product | | Kir1.1, ROMK1 | 11q24.3 | 3758 | ENSG00000151704 | OTTHUMG00000048247 | 276 | Potassium inwardly rectifying channel subfamily J |
| HGNC:6256 | KCNJ10 | potassium inwardly rectifying channel subfamily J member 10 | Approved | gene with protein product | | Kir4.1, Kir1.2 | 1q23.2 | 3766 | ENSG00000177807 | OTTHUMG00000024073 | 276 | Potassium inwardly rectifying channel subfamily J |
| HGNC:6257 | KCNJ11 | potassium inwardly rectifying channel subfamily J member 11 | Approved | gene with protein product | | Kir6.2, BIR | 11p15.1 | 3767 | ENSG00000187486 | OTTHUMG00000165914 | 276 | Potassium inwardly rectifying channel subfamily J |
| HGNC:6258 | KCNJ12 | potassium inwardly rectifying channel subfamily J member 12 | Approved | gene with protein product | KCNJN1 | Kir2.2, Kir2.2v, IRK2, hIRK1 | 17p11.2 | 3768 | ENSG00000184185 | OTTHUMG00000132039 | 276 | Potassium inwardly rectifying channel subfamily J |
| HGNC:6259 | KCNJ13 | potassium inwardly rectifying channel subfamily J member 13 | Approved | gene with protein product | | Kir7.1, Kir1.4, LCA16 | 2q37.1 | 3769 | ENSG00000115474 | OTTHUMG00000153292 | 276 | Potassium inwardly rectifying channel subfamily J |
| HGNC:6260 | KCNJ14 | potassium inwardly rectifying channel subfamily J member 14 | Approved | gene with protein product | | Kir2.4, IRK4 | 19q13.33 | 3770 | ENSG00000182324 | OTTHUMG00000183307 | 276 | Potassium inwardly rectifying channel subfamily J |
| HGNC:6261 | KCNJ15 | potassium inwardly rectifying channel subfamily J member 15 | Approved | gene with protein product | | Kir4.2, Kir1.3, IRKK | 21q22.13-q22.2 | 3772 | ENSG00000157551 | OTTHUMG00000090609 | 276 | Potassium inwardly rectifying channel subfamily J |
| HGNC:6262 | KCNJ16 | potassium inwardly rectifying channel subfamily J member 16 | Approved | gene with protein product | | Kir5.1, BIR9 | 17q24.3 | 3773 | ENSG00000153822 | OTTHUMG00000180349 | 276 | Potassium inwardly rectifying channel subfamily J |
| HGNC:39080 | KCNJ18 | potassium inwardly rectifying channel subfamily J member 18 | Approved | gene with protein product | | KIR2.6, TTPP2 | 17p11.2 | 1E+08 | ENSG00000260458 | OTTHUMG00000175863 | 276 | Potassium inwardly rectifying channel subfamily J |
| HGNC:6263 | KCNJ2 | potassium inwardly rectifying channel subfamily J member 2 | Approved | gene with protein product | | Kir2.1, IRK1, LQT7 | 17q24.3 | 3759 | ENSG00000123700 | OTTHUMG00000180351 | 276 | Potassium inwardly rectifying channel subfamily J |
| HGNC:6264 | KCNJ3 | potassium inwardly rectifying channel subfamily J member 3 | Approved | gene with protein product | | Kir3.1, GIRK1, KGA | 2q24.1 | 3760 | ENSG00000162989 | OTTHUMG00000131937 | 276 | Potassium inwardly rectifying channel subfamily J |
| HGNC:6265 | KCNJ4 | potassium inwardly rectifying channel subfamily J member 4 | Approved | gene with protein product | | Kir2.3, HIR, HRK1, hIRK2, IRK3 | 22q13.1 | 3761 | ENSG00000168135 | OTTHUMG00000151131 | 276 | Potassium inwardly rectifying channel subfamily J |
| HGNC:6266 | KCNJ5 | potassium inwardly rectifying channel subfamily J member 5 | Approved | gene with protein product | | Kir3.4, CIR, KATP1, GIRK4, LQT13 | 11q24.3 | 3762 | ENSG00000120457 | OTTHUMG00000165795 | 276 | Potassium inwardly rectifying channel subfamily J |
| HGNC:6267 | KCNJ6 | potassium inwardly rectifying channel subfamily J member 6 | Approved | gene with protein product | KCNJ7 | Kir3.2, GIRK2, KATP2, BIR1, hiGIRK2 | 21q22.13 | 3763 | ENSG00000157542 | OTTHUMG00000086667 | 276 | Potassium inwardly rectifying channel subfamily J |
| HGNC:6269 | KCNJ8 | potassium inwardly rectifying channel subfamily J member 8 | Approved | gene with protein product | | Kir6.1 | 12p12.1 | 3764 | ENSG00000121361 | OTTHUMG00000169093 | 276 | Potassium inwardly rectifying channel subfamily J |
| HGNC:6270 | KCNJ9 | potassium inwardly rectifying channel subfamily J member 9 | Approved | gene with protein product | | Kir3.3, GIRK3 | 1q23.2 | 3765 | ENSG00000162728 | OTTHUMG00000024072 | 276 | Potassium inwardly rectifying channel subfamily J |
| HGNC:18865 | KCNT1 | potassium sodium-activated channel subfamily T member 1 | Approved | gene with protein product | | KCa4.1, KIAA1422, SLACK, Slo2.2 | 9q34.3 | 57582 | ENSG00000107147 | OTTHUMG00000020917 | 856 | Potassium sodium-activated channel subfamily T |
| HGNC:18866 | KCNT2 | potassium sodium-activated channel subfamily T member 2 | Approved | gene with protein product | | KCa4.2, SLICK, SLO2.1 | 1q31.3 | 343450 | ENSG00000162687 | OTTHUMG00000035611 | 856 | Potassium sodium-activated channel subfamily T |
| HGNC:6272 | KCNK1 | potassium two pore domain channel subfamily K member 1 | Approved | gene with protein product | | K2p1.1, DPK, TWIK-1 | 1q42.2 | 3775 | ENSG00000135750 | OTTHUMG00000037923 | 277 | Potassium two pore domain channel subfamily K |
| HGNC:6273 | KCNK10 | potassium two pore domain channel subfamily K member 10 | Approved | gene with protein product | | K2p10.1, TREK-2, TREK2, PPP1R97 | 14q31.3 | 54207 | ENSG00000100433 | OTTHUMG00000170743 | 277 | Potassium two pore domain channel subfamily K |
| HGNC:6274 | KCNK12 | potassium two pore domain channel subfamily K member 12 | Approved | gene with protein product | | THIK-2, THIK2, K2p12.1 | 2p16.3 | 56660 | ENSG00000184261 | OTTHUMG00000129131 | 277 | Potassium two pore domain channel subfamily K |
| HGNC:6275 | KCNK13 | potassium two pore domain channel subfamily K member 13 | Approved | gene with protein product | | K2p13.1, THIK-1, THIK1 | 14q32.11 | 56659 | ENSG00000152315 | OTTHUMG00000171017 | 277 | Potassium two pore domain channel subfamily K |
| HGNC:13814 | KCNK15 | potassium two pore domain channel subfamily K member 15 | Approved | gene with protein product | KCNK11, KCNK14 | K2p15.1, dJ781B1.1, KT3.3, KIAA0237, TASK5, TASK-5 | 20q13.12 | 60598 | ENSG00000124249 | OTTHUMG00000032544 | 277 | Potassium two pore domain channel subfamily K |
| HGNC:14464 | KCNK16 | potassium two pore domain channel subfamily K member 16 | Approved | gene with protein product | | K2p16.1, TALK-1, TALK1 | 6p21.2 | 83795 | ENSG00000095981 | OTTHUMG00000014645 | 277 | Potassium two pore domain channel subfamily K |
| HGNC:14465 | KCNK17 | potassium two pore domain channel subfamily K member 17 | Approved | gene with protein product | | K2p17.1, TALK-2, TALK2, TASK4, TASK-4 | 6p21.2 | 89822 | ENSG00000124780 | OTTHUMG00000014646 | 277 | Potassium two pore domain channel subfamily K |
| HGNC:19439 | KCNK18 | potassium two pore domain channel subfamily K member 18 | Approved | gene with protein product | | K2p18.1, TRESK-2, TRESK2, TRESK, TRIK | 10q25.3 | 338567 | ENSG00000186795 | OTTHUMG00000019120 | 277 | Potassium two pore domain channel subfamily K |
| HGNC:6277 | KCNK2 | potassium two pore domain channel subfamily K member 2 | Approved | gene with protein product | | K2p2.1, TREK-1 | 1q41 | 3776 | ENSG00000082482 | OTTHUMG00000037017 | 277 | Potassium two pore domain channel subfamily K |
| HGNC:6278 | KCNK3 | potassium two pore domain channel subfamily K member 3 | Approved | gene with protein product | | K2p3.1, TASK, TASK-1, TASK1 | 2p23.3 | 3777 | ENSG00000171303 | OTTHUMG00000125530 | 277 | Potassium two pore domain channel subfamily K |
| HGNC:6279 | KCNK4 | potassium two pore domain channel subfamily K member 4 | Approved | gene with protein product | | K2p4.1, TRAAK | 11q13.1 | 50801 | ENSG00000182450 | OTTHUMG00000168006 | 277 | Potassium two pore domain channel subfamily K |
| HGNC:6280 | KCNK5 | potassium two pore domain channel subfamily K member 5 | Approved | gene with protein product | | K2p5.1, TASK-2, TASK2 | 6p21.2 | 8645 | ENSG00000164626 | OTTHUMG00000014642 | 277 | Potassium two pore domain channel subfamily K |
| HGNC:6281 | KCNK6 | potassium two pore domain channel subfamily K member 6 | Approved | gene with protein product | | K2p6.1, TWIK-2 | 19q13.2 | 9424 | ENSG00000099337 | OTTHUMG00000182325 | 277 | Potassium two pore domain channel subfamily K |
| HGNC:6282 | KCNK7 | potassium two pore domain channel subfamily K member 7 | Approved | gene with protein product | | K2p7.1 | 11q13.1 | 10089 | ENSG00000173338 | OTTHUMG00000166528 | 277 | Potassium two pore domain channel subfamily K |
| HGNC:6283 | KCNK9 | potassium two pore domain channel subfamily K member 9 | Approved | gene with protein product | | K2p9.1, TASK3, TASK-3 | 8q24.3 | 51305 | ENSG00000169427 | OTTHUMG00000164186 | 277 | Potassium two pore domain channel subfamily K |
| HGNC:6218 | KCNA1 | potassium voltage-gated channel subfamily A member 1 | Approved | gene with protein product | AEMK | Kv1.1, RBK1, HUK1, MBK1 | 12p13.32 | 3736 | ENSG00000111262 | OTTHUMG00000044398 | 274 | Potassium voltage-gated channels |
| HGNC:6219 | KCNA10 | potassium voltage-gated channel subfamily A member 10 | Approved | gene with protein product | | Kv1.8 | 1p13.3 | 3744 | ENSG00000143105 | OTTHUMG00000022785 | 274 | Potassium voltage-gated channels |
| HGNC:6220 | KCNA2 | potassium voltage-gated channel subfamily A member 2 | Approved | gene with protein product | | Kv1.2, HK4 | 1p13.3 | 3737 | ENSG00000177301 | OTTHUMG00000011567 | 274 | Potassium voltage-gated channels |
| HGNC:6221 | KCNA3 | potassium voltage-gated channel subfamily A member 3 | Approved | gene with protein product | | Kv1.3, MK3, HLK3, HPCN3, RP11-284N8.3 | 1p13.3 | 3738 | ENSG00000177272 | OTTHUMG00000034493 | 274 | Potassium voltage-gated channels |
| HGNC:6222 | KCNA4 | potassium voltage-gated channel subfamily A member 4 | Approved | gene with protein product | KCNA4L | Kv1.4, HK1, HPCN2, PCN2 | 11p14.1 | 3739 | ENSG00000182255 | OTTHUMG00000166144 | 274 | Potassium voltage-gated channels |
| HGNC:6224 | KCNA5 | potassium voltage-gated channel subfamily A member 5 | Approved | gene with protein product | | Kv1.5, HK2, HPCN1 | 12p13.32 | 3741 | ENSG00000130037 | OTTHUMG00000168232 | 274 | Potassium voltage-gated channels |
| HGNC:6225 | KCNA6 | potassium voltage-gated channel subfamily A member 6 | Approved | gene with protein product | | Kv1.6, HBK2, PPP1R96 | 12p13.32 | 3742 | ENSG00000151079 | OTTHUMG00000168225 | 274 | Potassium voltage-gated channels |
| HGNC:6226 | KCNA7 | potassium voltage-gated channel subfamily A member 7 | Approved | gene with protein product | | Kv1.7, HAK6 | 19q13.33 | 3743 | ENSG00000104848 | OTTHUMG00000183343 | 274 | Potassium voltage-gated channels |
| HGNC:6231 | KCNB1 | potassium voltage-gated channel subfamily B member 1 | Approved | gene with protein product | | Kv2.1 | 20q13.13 | 3745 | ENSG00000158445 | OTTHUMG00000033051 | 274 | Potassium voltage-gated channels |
| HGNC:6232 | KCNB2 | potassium voltage-gated channel subfamily B member 2 | Approved | gene with protein product | | Kv2.2 | 8q21.11 | 9312 | ENSG00000182674 | OTTHUMG00000164498 | 274 | Potassium voltage-gated channels |
| HGNC:6233 | KCNC1 | potassium voltage-gated channel subfamily C member 1 | Approved | gene with protein product | | Kv3.1 | 11p15.1 | 3746 | ENSG00000129159 | OTTHUMG00000166359 | 274 | Potassium voltage-gated channels |
| HGNC:6234 | KCNC2 | potassium voltage-gated channel subfamily C member 2 | Approved | gene with protein product | | Kv3.2 | 12q21.1 | 3747 | ENSG00000166006 | OTTHUMG00000169717 | 274 | Potassium voltage-gated channels |
| HGNC:6235 | KCNC3 | potassium voltage-gated channel subfamily C member 3 | Approved | gene with protein product | SCA13 | Kv3.3 | 19q13.33 | 3748 | ENSG00000131398 | OTTHUMG00000044580 | 274 | Potassium voltage-gated channels |
| HGNC:6236 | KCNC4 | potassium voltage-gated channel subfamily C member 4 | Approved | gene with protein product | C1orf30 | Kv3.4, HKSHIIIC | 1p13.3 | 3749 | ENSG00000116396 | OTTHUMG00000011037 | 274 | Potassium voltage-gated channels |
| HGNC:6237 | KCND1 | potassium voltage-gated channel subfamily D member 1 | Approved | gene with protein product | | Kv4.1 | Xp11.23 | 3750 | ENSG00000102057 | OTTHUMG00000024127 | 274 | Potassium voltage-gated channels |
| HGNC:6238 | KCND2 | potassium voltage-gated channel subfamily D member 2 | Approved | gene with protein product | | Kv4.2, RK5, KIAA1044 | 7q31.31 | 3751 | ENSG00000184408 | OTTHUMG00000156989 | 274 | Potassium voltage-gated channels |
| HGNC:6239 | KCND3 | potassium voltage-gated channel subfamily D member 3 | Approved | gene with protein product | SCA22, SCA19 | Kv4.3, KSHIVB | 1p13.2 | 3752 | ENSG00000171385 | OTTHUMG00000011989 | 274 | Potassium voltage-gated channels |
| HGNC:6246 | KCNF1 | potassium voltage-gated channel modifier subfamily F member 1 | Approved | gene with protein product | KCNF | Kv5.1, kH1, IK8 | 2p25.1 | 3754 | ENSG00000162975 | OTTHUMG00000119054 | 274 | Potassium voltage-gated channels |
| HGNC:6248 | KCNG1 | potassium voltage-gated channel modifier subfamily G member 1 | Approved | gene with protein product | KCNG | Kv6.1, kH2, K13 | 20q13.13 | 3755 | ENSG00000026559 | OTTHUMG00000032745 | 274 | Potassium voltage-gated channels |
| HGNC:6249 | KCNG2 | potassium voltage-gated channel modifier subfamily G member 2 | Approved | gene with protein product | | Kv6.2, KCNF2 | 18q23 | 26251 | ENSG00000178342 | OTTHUMG00000044541 | 274 | Potassium voltage-gated channels |
| HGNC:18306 | KCNG3 | potassium voltage-gated channel modifier subfamily G member 3 | Approved | gene with protein product | | Kv6.3 | 2p21 | 170850 | ENSG00000171126 | OTTHUMG00000128604 | 274 | Potassium voltage-gated channels |
| HGNC:19697 | KCNG4 | potassium voltage-gated channel modifier subfamily G member 4 | Approved | gene with protein product | | Kv6.4 | 16q24.1 | 93107 | ENSG00000168418 | OTTHUMG00000137638 | 274 | Potassium voltage-gated channels |
| HGNC:6250 | KCNH1 | potassium voltage-gated channel subfamily H member 1 | Approved | gene with protein product | | Kv10.1, eag, h-eag, eag1, hEAG, K(V)10.1 | 1q32.2 | 3756 | ENSG00000143473 | OTTHUMG00000036309 | 274 | Potassium voltage-gated channels |
| HGNC:6251 | KCNH2 | potassium voltage-gated channel subfamily H member 2 | Approved | gene with protein product | LQT2 | Kv11.1, HERG, erg1 | 7q36.1 | 3757 | ENSG00000055118 | OTTHUMG00000158341 | 274 | Potassium voltage-gated channels |
| HGNC:6252 | KCNH3 | potassium voltage-gated channel subfamily H member 3 | Approved | gene with protein product | | Kv12.2, BEC1, elk2 | 12q13.12 | 23416 | ENSG00000135519 | OTTHUMG00000169517 | 274 | Potassium voltage-gated channels |
| HGNC:6253 | KCNH4 | potassium voltage-gated channel subfamily H member 4 | Approved | gene with protein product | | Kv12.3, elk1 | 17q21.2 | 23415 | ENSG00000089558 | OTTHUMG00000180106 | 274 | Potassium voltage-gated channels |
| HGNC:6254 | KCNH5 | potassium voltage-gated channel subfamily H member 5 | Approved | gene with protein product | | Kv10.2, H-EAG2, eag2, hEAG2 | 14q23.2 | 27133 | ENSG00000140015 | OTTHUMG00000029041 | 274 | Potassium voltage-gated channels |
| HGNC:18862 | KCNH6 | potassium voltage-gated channel subfamily H member 6 | Approved | gene with protein product | | Kv11.2, erg2, HERG2 | 17q23.3 | 81033 | ENSG00000173826 | OTTHUMG00000178901 | 274 | Potassium voltage-gated channels |
| HGNC:18863 | KCNH7 | potassium voltage-gated channel subfamily H member 7 | Approved | gene with protein product | | Kv11.3, HERG3, erg3 | 2q24.2 | 90134 | ENSG00000184611 | OTTHUMG00000132069 | 274 | Potassium voltage-gated channels |
| HGNC:18864 | KCNH8 | potassium voltage-gated channel subfamily H member 8 | Approved | gene with protein product | | Kv12.1, elk3 | 3p24.3 | 131096 | ENSG00000183960 | OTTHUMG00000129891 | 274 | Potassium voltage-gated channels |
| HGNC:6294 | KCNQ1 | potassium voltage-gated channel subfamily Q member 1 | Approved | gene with protein product | LQT, KCNA9 | Kv7.1, KCNA8, KVLQT1, JLNS1, LQT1 | 11p15.5-p15.4 | 3784 | ENSG00000053918 | OTTHUMG00000009900 | 274 | Potassium voltage-gated channels |
| HGNC:6296 | KCNQ2 | potassium voltage-gated channel subfamily Q member 2 | Approved | gene with protein product | EBN, EBN1 | Kv7.2, ENB1, BFNC, KCNA11, HNSPC | 20q13.33 | 3785 | ENSG00000075043 | OTTHUMG00000033049 | 274 | Potassium voltage-gated channels |
| HGNC:6297 | KCNQ3 | potassium voltage-gated channel subfamily Q member 3 | Approved | gene with protein product | EBN2 | Kv7.3 | 8q24.22 | 3786 | ENSG00000184156 | OTTHUMG00000137472 | 274 | Potassium voltage-gated channels |
| HGNC:6298 | KCNQ4 | potassium voltage-gated channel subfamily Q member 4 | Approved | gene with protein product | DFNA2 | Kv7.4 | 1p34.2 | 9132 | ENSG00000117013 | OTTHUMG00000007730 | 274 | Potassium voltage-gated channels |
| HGNC:6299 | KCNQ5 | potassium voltage-gated channel subfamily Q member 5 | Approved | gene with protein product | | Kv7.5 | 6q13 | 56479 | ENSG00000185760 | OTTHUMG00000015020 | 274 | Potassium voltage-gated channels |
| HGNC:6300 | KCNS1 | potassium voltage-gated channel modifier subfamily S member 1 | Approved | gene with protein product | | Kv9.1 | 20q13.12 | 3787 | ENSG00000124134 | OTTHUMG00000033079 | 274 | Potassium voltage-gated channels |
| HGNC:6301 | KCNS2 | potassium voltage-gated channel modifier subfamily S member 2 | Approved | gene with protein product | | Kv9.2 | 8q22.2 | 3788 | ENSG00000156486 | OTTHUMG00000044337 | 274 | Potassium voltage-gated channels |
| HGNC:6302 | KCNS3 | potassium voltage-gated channel modifier subfamily S member 3 | Approved | gene with protein product | | Kv9.3 | 2p24.2 | 3790 | ENSG00000170745 | OTTHUMG00000044150 | 274 | Potassium voltage-gated channels |
| HGNC:18861 | KCNV1 | potassium voltage-gated channel modifier subfamily V member 1 | Approved | gene with protein product | | Kv8.1 | 8q23.2 | 27012 | ENSG00000164794 | OTTHUMG00000165637 | 274 | Potassium voltage-gated channels |
| HGNC:19698 | KCNV2 | potassium voltage-gated channel modifier subfamily V member 2 | Approved | gene with protein product | | Kv8.2 | 9p24.2 | 169522 | ENSG00000168263 | OTTHUMG00000019449 | 274 | Potassium voltage-gated channels |
| HGNC:8533 | P2RX1 | purinergic receptor P2X 1 | Approved | gene with protein product | | P2X1 | 17p13.2 | 5023 | ENSG00000108405 | OTTHUMG00000177673 | 214 | Purinergic receptors P2X |
| HGNC:15459 | P2RX2 | purinergic receptor P2X 2 | Approved | gene with protein product | DFNA41 | P2X2 | 12q24.33 | 22953 | ENSG00000187848 | OTTHUMG00000168018 | 214 | Purinergic receptors P2X |
| HGNC:8534 | P2RX3 | purinergic receptor P2X 3 | Approved | gene with protein product | | P2X3 | 11q12.1 | 5024 | ENSG00000109991 | OTTHUMG00000167025 | 214 | Purinergic receptors P2X |
| HGNC:8535 | P2RX4 | purinergic receptor P2X 4 | Approved | gene with protein product | | P2X4 | 12q24.31 | 5025 | ENSG00000135124 | OTTHUMG00000169155 | 214 | Purinergic receptors P2X |
| HGNC:8536 | P2RX5 | purinergic receptor P2X 5 | Approved | gene with protein product | | P2X5, LRH-1 | 17p13.2 | 5026 | ENSG00000083454 | OTTHUMG00000090700 | 214 | Purinergic receptors P2X |
| HGNC:8538 | P2RX6 | purinergic receptor P2X 6 | Approved | gene with protein product | P2RXL1 | P2XM, MGC129625, P2X6 | 22q11.21 | 9127 | ENSG00000099957 | OTTHUMG00000150689 | 214 | Purinergic receptors P2X |
| HGNC:8537 | P2RX7 | purinergic receptor P2X 7 | Approved | gene with protein product | | P2X7, MGC20089 | 12q24.31 | 5027 | ENSG00000089041 | OTTHUMG00000169153 | 214 | Purinergic receptors P2X |
| HGNC:10483 | RYR1 | ryanodine receptor 1 | Approved | gene with protein product | MHS, MHS1, CCO | RYR, PPP1R137 | 19q13.2 | 6261 | ENSG00000196218 | OTTHUMG00000182403 | 287 | Ryanodine receptors |
| HGNC:10484 | RYR2 | ryanodine receptor 2 | Approved | gene with protein product | ARVD2 | ARVC2, VTSIP | 1q43 | 6262 | ENSG00000198626 | OTTHUMG00000039543 | 287 | Ryanodine receptors |
| HGNC:10485 | RYR3 | ryanodine receptor 3 | Approved | gene with protein product | | | 15q13.3-q14 | 6263 | ENSG00000198838 | OTTHUMG00000172253 | 287 | Ryanodine receptors |
| HGNC:10599 | SCNN1A | sodium channel epithelial 1 subunit alpha | Approved | gene with protein product | SCNN1 | ENaCalpha | 12p13.31 | 6337 | ENSG00000111319 | OTTHUMG00000168268 | 185 | Sodium channels epithelial |
| HGNC:10600 | SCNN1B | sodium channel epithelial 1 subunit beta | Approved | gene with protein product | | ENaCbeta | 16p12.2 | 6338 | ENSG00000168447 | OTTHUMG00000131608 | 185 | Sodium channels epithelial |
| HGNC:10601 | SCNN1D | sodium channel epithelial 1 subunit delta | Approved | gene with protein product | | ENaCdelta, dNaCh | 1p36.33 | 6339 | ENSG00000162572 | OTTHUMG00000002081 | 185 | Sodium channels epithelial |
| HGNC:10602 | SCNN1G | sodium channel epithelial 1 subunit gamma | Approved | gene with protein product | | ENaCgamma, SCNEG | 16p12.2 | 6340 | ENSG00000166828 | OTTHUMG00000131609 | 185 | Sodium channels epithelial |
| HGNC:19082 | NALCN | sodium leak channel, non-selective | Approved | gene with protein product | VGCNL1 | bA430M15.1, CanIon | 13q32.3-q33.1 | 259232 | ENSG00000102452 | OTTHUMG00000017295 | 283 | Sodium leak channels, non selective |
| HGNC:10582 | SCN10A | sodium voltage-gated channel alpha subunit 10 | Approved | gene with protein product | | Nav1.8, hPN3, SNS, PN3 | 3p22.2 | 6336 | ENSG00000185313 | OTTHUMG00000048245 | 1203 | Sodium voltage-gated channel alpha subunits |
| HGNC:10583 | SCN11A | sodium voltage-gated channel alpha subunit 11 | Approved | gene with protein product | SCN12A | Nav1.9, NaN, SNS-2 | 3p22.2 | 11280 | ENSG00000168356 | OTTHUMG00000048246 | 1203 | Sodium voltage-gated channel alpha subunits |
| HGNC:10585 | SCN1A | sodium voltage-gated channel alpha subunit 1 | Approved | gene with protein product | SCN1, FEB3 | Nav1.1, GEFSP2, HBSCI, NAC1, SMEI | 2q24.3 | 6323 | ENSG00000144285 | OTTHUMG00000044173 | 1203 | Sodium voltage-gated channel alpha subunits |
| HGNC:10588 | SCN2A | sodium voltage-gated channel alpha subunit 2 | Approved | gene with protein product | SCN2A1, SCN2A2 | Nav1.2, HBSCII, HBSCI | 2q24.3 | 6326 | ENSG00000136531 | OTTHUMG00000044172 | 1203 | Sodium voltage-gated channel alpha subunits |
| HGNC:10590 | SCN3A | sodium voltage-gated channel alpha subunit 3 | Approved | gene with protein product | | Nav1.3 | 2q24.3 | 6328 | ENSG00000153253 | OTTHUMG00000044171 | 1203 | Sodium voltage-gated channel alpha subunits |
| HGNC:10591 | SCN4A | sodium voltage-gated channel alpha subunit 4 | Approved | gene with protein product | HYKPP | Nav1.4, HYPP, SkM1 | 17q23.3 | 6329 | ENSG00000007314 | OTTHUMG00000179046 | 1203 | Sodium voltage-gated channel alpha subunits |
| HGNC:10593 | SCN5A | sodium voltage-gated channel alpha subunit 5 | Approved | gene with protein product | CMD1E | Nav1.5, LQT3, HB1, HBBD, PFHB1, IVF, HB2, HH1, SSS1, CDCD2, CMPD2, ICCD | 3p22.2 | 6331 | ENSG00000183873 | OTTHUMG00000156166 | 1203 | Sodium voltage-gated channel alpha subunits |
| HGNC:10596 | SCN8A | sodium voltage-gated channel alpha subunit 8 | Approved | gene with protein product | MED | Nav1.6, NaCh6, PN4, CerIII, CIAT | 12q13.13 | 6334 | ENSG00000196876 | OTTHUMG00000169490 | 1203 | Sodium voltage-gated channel alpha subunits |
| HGNC:10597 | SCN9A | sodium voltage-gated channel alpha subunit 9 | Approved | gene with protein product | | Nav1.7, PN1, NE-NA, NENA, ETHA | 2q24.3 | 6335 | ENSG00000169432 | OTTHUMG00000154044 | 1203 | Sodium voltage-gated channel alpha subunits |
| HGNC:10586 | SCN1B | sodium voltage-gated channel beta subunit 1 | Approved | gene with protein product | | | 19q13.11 | 6324 | ENSG00000105711 | OTTHUMG00000182472 | 1204 | Sodium voltage-gated channel beta subunits |
| HGNC:10589 | SCN2B | sodium voltage-gated channel beta subunit 2 | Approved | gene with protein product | | | 11q23.3 | 6327 | ENSG00000149575 | OTTHUMG00000048248 | 1204 | Sodium voltage-gated channel beta subunits |
| HGNC:20665 | SCN3B | sodium voltage-gated channel beta subunit 3 | Approved | gene with protein product | | HSA243396, SCNB3 | 11q24.1 | 55800 | ENSG00000166257 | OTTHUMG00000166006 | 1204 | Sodium voltage-gated channel beta subunits |
| HGNC:10592 | SCN4B | sodium voltage-gated channel beta subunit 4 | Approved | gene with protein product | | LQT10 | 11q23.3 | 6330 | ENSG00000177098 | OTTHUMG00000166994 | 1204 | Sodium voltage-gated channel beta subunits |
| HGNC:497 | TRPA1 | transient receptor potential cation channel subfamily A member 1 | Approved | gene with protein product | ANKTM1 |  | 8q21.11 | 8989 | ENSG00000104321 | OTTHUMG00000164516 | 249 | Transient receptor potential cation channels |
| HGNC:12333 | TRPC1 | transient receptor potential cation channel subfamily C member 1 | Approved | gene with protein product | | HTRP-1 | 3q23 | 7220 | ENSG00000144935 | OTTHUMG00000159301 | 249 | Transient receptor potential cation channels |
| HGNC:12334 | TRPC2 | transient receptor potential cation channel subfamily C member 2 (pseudogene) | Approved | pseudogene | |  | 11p15.4 | 7221 | ENSG00000182048 | OTTHUMG00000011712 | 249 | Transient receptor potential cation channels |
| HGNC:12335 | TRPC3 | transient receptor potential cation channel subfamily C member 3 | Approved | gene with protein product | | | 4q27 | 7222 | ENSG00000138741 | OTTHUMG00000133069 | 249 | Transient receptor potential cation channels |
| HGNC:12336 | TRPC4 | transient receptor potential cation channel subfamily C member 4 | Approved | gene with protein product | | HTRP4, TRP4 | 13q13.3 | 7223 | ENSG00000133107 | OTTHUMG00000016752 | 249 | Transient receptor potential cation channels |
| HGNC:12337 | TRPC5 | transient receptor potential cation channel subfamily C member 5 | Approved | gene with protein product | | PPP1R159 | Xq23 | 7224 | ENSG00000072315 | OTTHUMG00000022212 | 249 | Transient receptor potential cation channels |
| HGNC:12338 | TRPC6 | transient receptor potential cation channel subfamily C member 6 | Approved | gene with protein product | FSGS2 | TRP6 | 11q22.1 | 7225 | ENSG00000137672 | OTTHUMG00000167483 | 249 | Transient receptor potential cation channels |
| HGNC:20754 | TRPC7 | transient receptor potential cation channel subfamily C member 7 | Approved | gene with protein product | | | 5q31.1 | 57113 | ENSG00000069018 | OTTHUMG00000189265 | 249 | Transient receptor potential cation channels |
| HGNC:7146 | TRPM1 | transient receptor potential cation channel subfamily M member 1 | Approved | gene with protein product | MLSN1 | LTRPC1, CSNB1C | 15q13.3 | 4308 | ENSG00000134160 | OTTHUMG00000129267 | 249 | Transient receptor potential cation channels |
| HGNC:12339 | TRPM2 | transient receptor potential cation channel subfamily M member 2 | Approved | gene with protein product | TRPC7 | KNP3, LTRPC2, NUDT9L1, NUDT9H, EREG1 | 21q22.3 | 7226 | ENSG00000142185 | OTTHUMG00000040840 | 249 | Transient receptor potential cation channels |
| HGNC:17992 | TRPM3 | transient receptor potential cation channel subfamily M member 3 | Approved | gene with protein product | | KIAA1616, LTRPC3, GON-2 | 9q21.12-q21.13 | 80036 | ENSG00000083067 | OTTHUMG00000019997 | 249 | Transient receptor potential cation channels |
| HGNC:17993 | TRPM4 | transient receptor potential cation channel subfamily M member 4 | Approved | gene with protein product | | FLJ20041 | 19q13.33 | 54795 | ENSG00000130529 | OTTHUMG00000183184 | 249 | Transient receptor potential cation channels |
| HGNC:14323 | TRPM5 | transient receptor potential cation channel subfamily M member 5 | Approved | gene with protein product | | LTRPC5, MTR1 | 11p15.5 | 29850 | ENSG00000070985 | OTTHUMG00000009896 | 249 | Transient receptor potential cation channels |
| HGNC:17995 | TRPM6 | transient receptor potential cation channel subfamily M member 6 | Approved | gene with protein product | HOMG, HSH | CHAK2, FLJ22628 | 9q21.13 | 140803 | ENSG00000119121 | OTTHUMG00000020027 | 249 | Transient receptor potential cation channels |
| HGNC:17994 | TRPM7 | transient receptor potential cation channel subfamily M member 7 | Approved | gene with protein product | | CHAK1, LTRPC7, TRP-PLIK | 15q21.2 | 54822 | ENSG00000092439 | OTTHUMG00000172449 | 249 | Transient receptor potential cation channels |
| HGNC:17961 | TRPM8 | transient receptor potential cation channel subfamily M member 8 | Approved | gene with protein product | | | 2q37.1 | 79054 | ENSG00000144481 | OTTHUMG00000059129 | 249 | Transient receptor potential cation channels |
| HGNC:13356 | MCOLN1 | mucolipin TRP cation channel 1 | Approved | gene with protein product | | TRPML1, ML4, MLIV, MST080, MSTP080, TRPM-L1 | 19p13.2 | 57192 | ENSG00000090674 | OTTHUMG00000182046 | 249 | Transient receptor potential cation channels |
| HGNC:13357 | MCOLN2 | mucolipin TRP cation channel 2 | Approved | gene with protein product | | TRPML2, FLJ36691, TRP-ML2 | 1p22.3 | 255231 | ENSG00000153898 | OTTHUMG00000009954 | 249 | Transient receptor potential cation channels |
| HGNC:13358 | MCOLN3 | mucolipin TRP cation channel 3 | Approved | gene with protein product | | TRPML3, FLJ11006, TRP-ML3 | 1p22.3 | 55283 | ENSG00000055732 | OTTHUMG00000009955 | 249 | Transient receptor potential cation channels |
| HGNC:9009 | PKD2 | polycystin 2, transient receptor potential cation channel | Approved | gene with protein product | | PKD4, PC2, Pc-2, TRPP2 | 4q22.1 | 5311 | ENSG00000118762 | OTTHUMG00000160982 | 249 | Transient receptor potential cation channels |
| HGNC:9011 | PKD2L1 | polycystin 2 like 1, transient receptor potential cation channel | Approved | gene with protein product | PKD2L, PKDL | PCL, TRPP3 | 10q24.31 | 9033 | ENSG00000107593 | OTTHUMG00000018910 | 249 | Transient receptor potential cation channels |
| HGNC:9012 | PKD2L2 | polycystin 2 like 2, transient receptor potential cation channel | Approved | gene with protein product | | TRPP5 | 5q31.2 | 27039 | ENSG00000078795 | OTTHUMG00000163306 | 249 | Transient receptor potential cation channels |
| HGNC:12716 | TRPV1 | transient receptor potential cation channel subfamily V member 1 | Approved | gene with protein product | VR1 |  | 17p13.2 | 7442 | ENSG00000196689 | OTTHUMG00000177649 | 249 | Transient receptor potential cation channels |
| HGNC:18082 | TRPV2 | transient receptor potential cation channel subfamily V member 2 | Approved | gene with protein product | | VRL, VRL-1, VRL1 | 17p11.2 | 51393 | ENSG00000187688 | OTTHUMG00000058989 | 249 | Transient receptor potential cation channels |
| HGNC:18084 | TRPV3 | transient receptor potential cation channel subfamily V member 3 | Approved | gene with protein product | | VRL3 | 17p13.2 | 162514 | ENSG00000167723 | OTTHUMG00000090695 | 249 | Transient receptor potential cation channels |
| HGNC:18083 | TRPV4 | transient receptor potential cation channel subfamily V member 4 | Approved | gene with protein product | | OTRPC4, TRP12, VROAC, VRL-2, VR-OAC, CMT2C | 12q24.11 | 59341 | ENSG00000111199 | OTTHUMG00000169277 | 249 | Transient receptor potential cation channels |
| HGNC:3145 | TRPV5 | transient receptor potential cation channel subfamily V member 5 | Approved | gene with protein product | ECAC1 | CaT2 | 7q34 | 56302 | ENSG00000127412 | OTTHUMG00000157157 | 249 | Transient receptor potential cation channels |
| HGNC:14006 | TRPV6 | transient receptor potential cation channel subfamily V member 6 | Approved | gene with protein product | ECAC2 | CaT1 | 7q34 | 55503 | ENSG00000165125 | OTTHUMG00000157158 | 249 | Transient receptor potential cation channels |
| HGNC:18182 | TPCN1 | two pore segment channel 1 | Approved | gene with protein product | | KIAA1169, FLJ20612, TPC1 | 12q24.13 | 53373 | ENSG00000186815 | OTTHUMG00000169625 | 247 | Two pore segment channels |
| HGNC:20820 | TPCN2 | two pore segment channel 2 | Approved | gene with protein product | | TPC2 | 11q13.3 | 219931 | ENSG00000162341 | OTTHUMG00000167898 | 247 | Two pore segment channels |
| HGNC:12669 | VDAC1 | voltage dependent anion channel 1 | Approved | gene with protein product | | MGC111064, PORIN | 5q31.1 | 7416 | ENSG00000213585 | OTTHUMG00000129118 | 306 | Voltage dependent anion channels |
| HGNC:12672 | VDAC2 | voltage dependent anion channel 2 | Approved | gene with protein product | | | 10q22.2 | 7417 | ENSG00000165637 | OTTHUMG00000018517 | 306 | Voltage dependent anion channels |
| HGNC:12674 | VDAC3 | voltage dependent anion channel 3 | Approved | gene with protein product | | HD-VDAC3 | 8p11.21 | 7419 | ENSG00000078668 | OTTHUMG00000164168 | 306 | Voltage dependent anion channels |
| HGNC:19027 | LRRC8A | leucine rich repeat containing 8 VRAC subunit A | Approved | gene with protein product | LRRC8 | KIAA1437, FLJ10337, SWELL1 | 9q34.11 | 56262 | ENSG00000136802 | OTTHUMG00000020766 | 1158 | Volume regulated anion channel subunits |
| HGNC:30692 | LRRC8B | leucine rich repeat containing 8 VRAC subunit B | Approved | gene with protein product | | TA-LRRP, KIAA0231 | 1p22.2 | 23507 | ENSG00000197147 | OTTHUMG00000010129 | 1158 | Volume regulated anion channel subunits |
| HGNC:25075 | LRRC8C | leucine rich repeat containing 8 VRAC subunit C | Approved | gene with protein product | | AD158 | 1p22.2 | 84230 | ENSG00000171488 | OTTHUMG00000010305 | 1158 | Volume regulated anion channel subunits |
| HGNC:16992 | LRRC8D | leucine rich repeat containing 8 VRAC subunit D | Approved | gene with protein product | LRRC5 | FLJ10470 | 1p22.2 | 55144 | ENSG00000171492 | OTTHUMG00000010583 | 1158 | Volume regulated anion channel subunits |
| HGNC:26272 | LRRC8E | leucine rich repeat containing 8 VRAC subunit E | Approved | gene with protein product | | FLJ23420 | 19p13.2 | 80131 | ENSG00000171017 | OTTHUMG00000182455 | 1158 | Volume regulated anion channel subunits |
| HGNC:29504 | ZACN | zinc activated ion channel | Approved | gene with protein product | LGICZ1 | LGICZ, L2, ZAC, ZAC1 | 17q25.1 | 353174 | ENSG00000186919 | OTTHUMG00000157186 | 176 | Zinc activated channels |
