# Supplementary material for: Using eQTL Mendelian randomization and transcriptomic analysis to identify the relationship between ion channel genes and intracranial aneurysmal subarachnoid hemorrhage
Source: Medicine (Baltimore). 2025 May 16;104(20):e42457. doi: 10.1097/MD.0000000000042457 (PMC12091597; doi:10.1097/MD.0000000000042457)
Supplement: Supplementary file 2 [file medi-104-e42457-s002.docx]

| **Table S2 172 Ion Channel Genes with eQTLs in GWAS Summary Data** | | | | | | | | | | | | | | | | | | | | | |
| --- | --- | --- | --- | --- | --- | --- | --- | --- | --- | --- | --- | --- | --- | --- | --- | --- | --- | --- | --- | --- | --- |
| id | trait | ncase | group_name | year | author | consortium | sex | pmid | population | unit | sample_size | build | ncontrol | category | subcategory | ontology | note | mr | nsnp | priority | sd |
| eqtl-a-ENSG00000120903 | ENSG00000120903 | NA | public | 2018 | Vosa U | NA | Males and Females | NA | European | NA | 31684 | HG19/GRCh37 | NA | NA | NA | NA | NA | 1 | 20284 | 0 | NA |
| eqtl-a-ENSG00000165269 | ENSG00000165269 | NA | public | 2018 | Vosa U | NA | Males and Females | NA | European | NA | 23770 | HG19/GRCh37 | NA | NA | NA | NA | NA | 1 | 18010 | 0 | NA |
| eqtl-a-ENSG00000108405 | ENSG00000108405 | NA | public | 2018 | Vosa U | NA | Males and Females | NA | European | NA | 31684 | HG19/GRCh37 | NA | NA | NA | NA | NA | 1 | 20658 | 0 | NA |
| eqtl-a-ENSG00000127325 | ENSG00000127325 | NA | public | 2018 | Vosa U | NA | Males and Females | NA | European | NA | 29726 | HG19/GRCh37 | NA | NA | NA | NA | NA | 1 | 19158 | 0 | NA |
| eqtl-a-ENSG00000160716 | ENSG00000160716 | NA | public | 2018 | Vosa U | NA | Males and Females | NA | European | NA | 30137 | HG19/GRCh37 | NA | NA | NA | NA | NA | 1 | 15701 | 0 | NA |
| eqtl-a-ENSG00000161509 | ENSG00000161509 | NA | public | 2018 | Vosa U | NA | Males and Females | NA | European | NA | 30962 | HG19/GRCh37 | NA | NA | NA | NA | NA | 1 | 19088 | 0 | NA |
| eqtl-a-ENSG00000151079 | ENSG00000151079 | NA | public | 2018 | Vosa U | NA | Males and Females | NA | European | NA | 31684 | HG19/GRCh37 | NA | NA | NA | NA | NA | 1 | 18683 | 0 | NA |
| eqtl-a-ENSG00000121742 | ENSG00000121742 | NA | public | 2018 | Vosa U | NA | Males and Females | NA | European | NA | 31086 | HG19/GRCh37 | NA | NA | NA | NA | NA | 1 | 19898 | 0 | NA |
| eqtl-a-ENSG00000162341 | ENSG00000162341 | NA | public | 2018 | Vosa U | NA | Males and Females | NA | European | NA | 31684 | HG19/GRCh37 | NA | NA | NA | NA | NA | 1 | 18275 | 0 | NA |
| eqtl-a-ENSG00000187688 | ENSG00000187688 | NA | public | 2018 | Vosa U | NA | Males and Females | NA | European | NA | 31470 | HG19/GRCh37 | NA | NA | NA | NA | NA | 1 | 17917 | 0 | NA |
| eqtl-a-ENSG00000182389 | ENSG00000182389 | NA | public | 2018 | Vosa U | NA | Males and Females | NA | European | NA | 31684 | HG19/GRCh37 | NA | NA | NA | NA | NA | 1 | 18191 | 0 | NA |
| eqtl-a-ENSG00000078668 | ENSG00000078668 | NA | public | 2018 | Vosa U | NA | Males and Females | NA | European | NA | 31684 | HG19/GRCh37 | NA | NA | NA | NA | NA | 1 | 17549 | 0 | NA |
| eqtl-a-ENSG00000171492 | ENSG00000171492 | NA | public | 2018 | Vosa U | NA | Males and Females | NA | European | NA | 31470 | HG19/GRCh37 | NA | NA | NA | NA | NA | 1 | 17477 | 0 | NA |
| eqtl-a-ENSG00000114859 | ENSG00000114859 | NA | public | 2018 | Vosa U | NA | Males and Females | NA | European | NA | 14263 | HG19/GRCh37 | NA | NA | NA | NA | NA | 1 | 17797 | 0 | NA |
| eqtl-a-ENSG00000142408 | ENSG00000142408 | NA | public | 2018 | Vosa U | NA | Males and Females | NA | European | NA | 31470 | HG19/GRCh37 | NA | NA | NA | NA | NA | 1 | 23006 | 0 | NA |
| eqtl-a-ENSG00000067191 | ENSG00000067191 | NA | public | 2018 | Vosa U | NA | Males and Females | NA | European | NA | 31470 | HG19/GRCh37 | NA | NA | NA | NA | NA | 1 | 17257 | 0 | NA |
| eqtl-a-ENSG00000099957 | ENSG00000099957 | NA | public | 2018 | Vosa U | NA | Males and Females | NA | European | NA | 26196 | HG19/GRCh37 | NA | NA | NA | NA | NA | 1 | 15644 | 0 | NA |
| eqtl-a-ENSG00000142185 | ENSG00000142185 | NA | public | 2018 | Vosa U | NA | Males and Females | NA | European | NA | 31470 | HG19/GRCh37 | NA | NA | NA | NA | NA | 1 | 20686 | 0 | NA |
| eqtl-a-ENSG00000171488 | ENSG00000171488 | NA | public | 2018 | Vosa U | NA | Males and Females | NA | European | NA | 31470 | HG19/GRCh37 | NA | NA | NA | NA | NA | 1 | 17375 | 0 | NA |
| eqtl-a-ENSG00000198835 | ENSG00000198835 | NA | public | 2018 | Vosa U | NA | Males and Females | NA | European | NA | 31684 | HG19/GRCh37 | NA | NA | NA | NA | NA | 1 | 19241 | 0 | NA |
| eqtl-a-ENSG00000185101 | ENSG00000185101 | NA | public | 2018 | Vosa U | NA | Males and Females | NA | European | NA | 26181 | HG19/GRCh37 | NA | NA | NA | NA | NA | 1 | 16517 | 0 | NA |
| eqtl-a-ENSG00000007402 | ENSG00000007402 | NA | public | 2018 | Vosa U | NA | Males and Females | NA | European | NA | 31684 | HG19/GRCh37 | NA | NA | NA | NA | NA | 1 | 15273 | 0 | NA |
| eqtl-a-ENSG00000175294 | ENSG00000175294 | NA | public | 2018 | Vosa U | NA | Males and Females | NA | European | NA | 31684 | HG19/GRCh37 | NA | NA | NA | NA | NA | 1 | 16025 | 0 | NA |
| eqtl-a-ENSG00000111199 | ENSG00000111199 | NA | public | 2018 | Vosa U | NA | Males and Females | NA | European | NA | 31470 | HG19/GRCh37 | NA | NA | NA | NA | NA | 1 | 17401 | 0 | NA |
| eqtl-a-ENSG00000179397 | ENSG00000179397 | NA | public | 2018 | Vosa U | NA | Males and Females | NA | European | NA | 14263 | HG19/GRCh37 | NA | NA | NA | NA | NA | 1 | 17762 | 0 | NA |
| eqtl-a-ENSG00000119121 | ENSG00000119121 | NA | public | 2018 | Vosa U | NA | Males and Females | NA | European | NA | 31470 | HG19/GRCh37 | NA | NA | NA | NA | NA | 1 | 18593 | 0 | NA |
| eqtl-a-ENSG00000130037 | ENSG00000130037 | NA | public | 2018 | Vosa U | NA | Males and Females | NA | European | NA | 31346 | HG19/GRCh37 | NA | NA | NA | NA | NA | 1 | 17881 | 0 | NA |
| eqtl-a-ENSG00000144935 | ENSG00000144935 | NA | public | 2018 | Vosa U | NA | Males and Females | NA | European | NA | 31684 | HG19/GRCh37 | NA | NA | NA | NA | NA | 1 | 18299 | 0 | NA |
| eqtl-a-ENSG00000089041 | ENSG00000089041 | NA | public | 2018 | Vosa U | NA | Males and Females | NA | European | NA | 31684 | HG19/GRCh37 | NA | NA | NA | NA | NA | 1 | 18085 | 0 | NA |
| eqtl-a-ENSG00000184611 | ENSG00000184611 | NA | public | 2018 | Vosa U | NA | Males and Females | NA | European | NA | 26101 | HG19/GRCh37 | NA | NA | NA | NA | NA | 1 | 16957 | 0 | NA |
| eqtl-a-ENSG00000124780 | ENSG00000124780 | NA | public | 2018 | Vosa U | NA | Males and Females | NA | European | NA | 31684 | HG19/GRCh37 | NA | NA | NA | NA | NA | 1 | 19216 | 0 | NA |
| eqtl-a-ENSG00000074855 | ENSG00000074855 | NA | public | 2018 | Vosa U | NA | Males and Females | NA | European | NA | 26609 | HG19/GRCh37 | NA | NA | NA | NA | NA | 1 | 18648 | 0 | NA |
| eqtl-a-ENSG00000151067 | ENSG00000151067 | NA | public | 2018 | Vosa U | NA | Males and Females | NA | European | NA | 30748 | HG19/GRCh37 | NA | NA | NA | NA | NA | 1 | 19422 | 0 | NA |
| eqtl-a-ENSG00000162572 | ENSG00000162572 | NA | public | 2018 | Vosa U | NA | Males and Females | NA | European | NA | 31684 | HG19/GRCh37 | NA | NA | NA | NA | NA | 1 | 17222 | 0 | NA |
| eqtl-a-ENSG00000118762 | ENSG00000118762 | NA | public | 2018 | Vosa U | NA | Males and Females | NA | European | NA | 31470 | HG19/GRCh37 | NA | NA | NA | NA | NA | 1 | 18197 | 0 | NA |
| eqtl-a-ENSG00000104783 | ENSG00000104783 | NA | public | 2018 | Vosa U | NA | Males and Females | NA | European | NA | 31470 | HG19/GRCh37 | NA | NA | NA | NA | NA | 1 | 20536 | 0 | NA |
| eqtl-a-ENSG00000167995 | ENSG00000167995 | NA | public | 2018 | Vosa U | NA | Males and Females | NA | European | NA | 31684 | HG19/GRCh37 | NA | NA | NA | NA | NA | 1 | 17317 | 0 | NA |
| eqtl-a-ENSG00000177098 | ENSG00000177098 | NA | public | 2018 | Vosa U | NA | Males and Females | NA | European | NA | 31684 | HG19/GRCh37 | NA | NA | NA | NA | NA | 1 | 18709 | 0 | NA |
| eqtl-a-ENSG00000138741 | ENSG00000138741 | NA | public | 2018 | Vosa U | NA | Males and Females | NA | European | NA | 30551 | HG19/GRCh37 | NA | NA | NA | NA | NA | 1 | 19250 | 0 | NA |
| eqtl-a-ENSG00000152705 | ENSG00000152705 | NA | public | 2018 | Vosa U | NA | Males and Females | NA | European | NA | 30859 | HG19/GRCh37 | NA | NA | NA | NA | NA | 1 | 17752 | 0 | NA |
| eqtl-a-ENSG00000070729 | ENSG00000070729 | NA | public | 2018 | Vosa U | NA | Males and Females | NA | European | NA | 23944 | HG19/GRCh37 | NA | NA | NA | NA | NA | 1 | 19959 | 0 | NA |
| eqtl-a-ENSG00000141837 | ENSG00000141837 | NA | public | 2018 | Vosa U | NA | Males and Females | NA | European | NA | 31256 | HG19/GRCh37 | NA | NA | NA | NA | NA | 1 | 17681 | 0 | NA |
| eqtl-a-ENSG00000178301 | ENSG00000178301 | NA | public | 2018 | Vosa U | NA | Males and Females | NA | European | NA | 31684 | HG19/GRCh37 | NA | NA | NA | NA | NA | 1 | 18167 | 0 | NA |
| eqtl-a-ENSG00000127412 | ENSG00000127412 | NA | public | 2018 | Vosa U | NA | Males and Females | NA | European | NA | 8680 | HG19/GRCh37 | NA | NA | NA | NA | NA | 1 | 15802 | 0 | NA |
| eqtl-a-ENSG00000165637 | ENSG00000165637 | NA | public | 2018 | Vosa U | NA | Males and Females | NA | European | NA | 28703 | HG19/GRCh37 | NA | NA | NA | NA | NA | 1 | 16131 | 0 | NA |
| eqtl-a-ENSG00000055732 | ENSG00000055732 | NA | public | 2018 | Vosa U | NA | Males and Females | NA | European | NA | 31470 | HG19/GRCh37 | NA | NA | NA | NA | NA | 1 | 18384 | 0 | NA |
| eqtl-a-ENSG00000171189 | ENSG00000171189 | NA | public | 2018 | Vosa U | NA | Males and Females | NA | European | NA | 31176 | HG19/GRCh37 | NA | NA | NA | NA | NA | 1 | 18668 | 0 | NA |
| eqtl-a-ENSG00000213585 | ENSG00000213585 | NA | public | 2018 | Vosa U | NA | Males and Females | NA | European | NA | 11496 | HG19/GRCh37 | NA | NA | NA | NA | NA | 1 | 16729 | 0 | NA |
| eqtl-a-ENSG00000083454 | ENSG00000083454 | NA | public | 2018 | Vosa U | NA | Males and Females | NA | European | NA | 31470 | HG19/GRCh37 | NA | NA | NA | NA | NA | 1 | 20663 | 0 | NA |
| eqtl-a-ENSG00000152315 | ENSG00000152315 | NA | public | 2018 | Vosa U | NA | Males and Females | NA | European | NA | 31684 | HG19/GRCh37 | NA | NA | NA | NA | NA | 1 | 18870 | 0 | NA |
| eqtl-a-ENSG00000185760 | ENSG00000185760 | NA | public | 2018 | Vosa U | NA | Males and Females | NA | European | NA | 31256 | HG19/GRCh37 | NA | NA | NA | NA | NA | 1 | 18192 | 0 | NA |
| eqtl-a-ENSG00000149305 | ENSG00000149305 | NA | public | 2018 | Vosa U | NA | Males and Females | NA | European | NA | 28880 | HG19/GRCh37 | NA | NA | NA | NA | NA | 1 | 17396 | 0 | NA |
| eqtl-a-ENSG00000213199 | ENSG00000213199 | NA | public | 2018 | Vosa U | NA | Males and Females | NA | European | NA | 8269 | HG19/GRCh37 | NA | NA | NA | NA | NA | 1 | 18535 | 0 | NA |
| eqtl-a-ENSG00000078795 | ENSG00000078795 | NA | public | 2018 | Vosa U | NA | Males and Females | NA | European | NA | 30043 | HG19/GRCh37 | NA | NA | NA | NA | NA | 1 | 16616 | 0 | NA |
| eqtl-a-ENSG00000130433 | ENSG00000130433 | NA | public | 2018 | Vosa U | NA | Males and Females | NA | European | NA | 31684 | HG19/GRCh37 | NA | NA | NA | NA | NA | 1 | 23042 | 0 | NA |
| eqtl-a-ENSG00000170175 | ENSG00000170175 | NA | public | 2018 | Vosa U | NA | Males and Females | NA | European | NA | 31684 | HG19/GRCh37 | NA | NA | NA | NA | NA | 1 | 18789 | 0 | NA |
| eqtl-a-ENSG00000198838 | ENSG00000198838 | NA | public | 2018 | Vosa U | NA | Males and Females | NA | European | NA | 31470 | HG19/GRCh37 | NA | NA | NA | NA | NA | 1 | 20619 | 0 | NA |
| eqtl-a-ENSG00000157551 | ENSG00000157551 | NA | public | 2018 | Vosa U | NA | Males and Females | NA | European | NA | 31470 | HG19/GRCh37 | NA | NA | NA | NA | NA | 1 | 18752 | 0 | NA |
| eqtl-a-ENSG00000165125 | ENSG00000165125 | NA | public | 2018 | Vosa U | NA | Males and Females | NA | European | NA | 14263 | HG19/GRCh37 | NA | NA | NA | NA | NA | 1 | 16321 | 0 | NA |
| eqtl-a-ENSG00000183873 | ENSG00000183873 | NA | public | 2018 | Vosa U | NA | Males and Females | NA | European | NA | 26022 | HG19/GRCh37 | NA | NA | NA | NA | NA | 1 | 18167 | 0 | NA |
| eqtl-a-ENSG00000143603 | ENSG00000143603 | NA | public | 2018 | Vosa U | NA | Males and Females | NA | European | NA | 31684 | HG19/GRCh37 | NA | NA | NA | NA | NA | 1 | 15605 | 0 | NA |
| eqtl-a-ENSG00000187730 | ENSG00000187730 | NA | public | 2018 | Vosa U | NA | Males and Females | NA | European | NA | 30859 | HG19/GRCh37 | NA | NA | NA | NA | NA | 1 | 19374 | 0 | NA |
| eqtl-a-ENSG00000186919 | ENSG00000186919 | NA | public | 2018 | Vosa U | NA | Males and Females | NA | European | NA | 7106 | HG19/GRCh37 | NA | NA | NA | NA | NA | 1 | 16780 | 0 | NA |
| eqtl-a-ENSG00000007314 | ENSG00000007314 | NA | public | 2018 | Vosa U | NA | Males and Females | NA | European | NA | 31684 | HG19/GRCh37 | NA | NA | NA | NA | NA | 1 | 15725 | 0 | NA |
| eqtl-a-ENSG00000149575 | ENSG00000149575 | NA | public | 2018 | Vosa U | NA | Males and Females | NA | European | NA | 26101 | HG19/GRCh37 | NA | NA | NA | NA | NA | 1 | 18485 | 0 | NA |
| eqtl-a-ENSG00000240583 | ENSG00000240583 | NA | public | 2018 | Vosa U | NA | Males and Females | NA | European | NA | 31684 | HG19/GRCh37 | NA | NA | NA | NA | NA | 1 | 18314 | 0 | NA |
| eqtl-a-ENSG00000112782 | ENSG00000112782 | NA | public | 2018 | Vosa U | NA | Males and Females | NA | European | NA | 31684 | HG19/GRCh37 | NA | NA | NA | NA | NA | 1 | 18072 | 0 | NA |
| eqtl-a-ENSG00000198785 | ENSG00000198785 | NA | public | 2018 | Vosa U | NA | Males and Females | NA | European | NA | 31684 | HG19/GRCh37 | NA | NA | NA | NA | NA | 1 | 20199 | 0 | NA |
| eqtl-a-ENSG00000196557 | ENSG00000196557 | NA | public | 2018 | Vosa U | NA | Males and Females | NA | European | NA | 31470 | HG19/GRCh37 | NA | NA | NA | NA | NA | 1 | 21942 | 0 | NA |
| eqtl-a-ENSG00000105737 | ENSG00000105737 | NA | public | 2018 | Vosa U | NA | Males and Females | NA | European | NA | 31684 | HG19/GRCh37 | NA | NA | NA | NA | NA | 1 | 17788 | 0 | NA |
| eqtl-a-ENSG00000099338 | ENSG00000099338 | NA | public | 2018 | Vosa U | NA | Males and Females | NA | European | NA | 31684 | HG19/GRCh37 | NA | NA | NA | NA | NA | 1 | 18208 | 0 | NA |
| eqtl-a-ENSG00000182324 | ENSG00000182324 | NA | public | 2018 | Vosa U | NA | Males and Females | NA | European | NA | 31684 | HG19/GRCh37 | NA | NA | NA | NA | NA | 1 | 19762 | 0 | NA |
| eqtl-a-ENSG00000117013 | ENSG00000117013 | NA | public | 2018 | Vosa U | NA | Males and Females | NA | European | NA | 31470 | HG19/GRCh37 | NA | NA | NA | NA | NA | 1 | 18297 | 0 | NA |
| eqtl-a-ENSG00000166736 | ENSG00000166736 | NA | public | 2018 | Vosa U | NA | Males and Females | NA | European | NA | 31470 | HG19/GRCh37 | NA | NA | NA | NA | NA | 1 | 17999 | 0 | NA |
| eqtl-a-ENSG00000143630 | ENSG00000143630 | NA | public | 2018 | Vosa U | NA | Males and Females | NA | European | NA | 14263 | HG19/GRCh37 | NA | NA | NA | NA | NA | 1 | 15144 | 0 | NA |
| eqtl-a-ENSG00000187513 | ENSG00000187513 | NA | public | 2018 | Vosa U | NA | Males and Females | NA | European | NA | 30859 | HG19/GRCh37 | NA | NA | NA | NA | NA | 1 | 17926 | 0 | NA |
| eqtl-a-ENSG00000089558 | ENSG00000089558 | NA | public | 2018 | Vosa U | NA | Males and Females | NA | European | NA | 29218 | HG19/GRCh37 | NA | NA | NA | NA | NA | 1 | 16748 | 0 | NA |
| eqtl-a-ENSG00000160746 | ENSG00000160746 | NA | public | 2018 | Vosa U | NA | Males and Females | NA | European | NA | 26609 | HG19/GRCh37 | NA | NA | NA | NA | NA | 1 | 16596 | 0 | NA |
| eqtl-a-ENSG00000198216 | ENSG00000198216 | NA | public | 2018 | Vosa U | NA | Males and Females | NA | European | NA | 31684 | HG19/GRCh37 | NA | NA | NA | NA | NA | 1 | 18413 | 0 | NA |
| eqtl-a-ENSG00000167535 | ENSG00000167535 | NA | public | 2018 | Vosa U | NA | Males and Females | NA | European | NA | 31470 | HG19/GRCh37 | NA | NA | NA | NA | NA | 1 | 17505 | 0 | NA |
| eqtl-a-ENSG00000099337 | ENSG00000099337 | NA | public | 2018 | Vosa U | NA | Males and Females | NA | European | NA | 31644 | HG19/GRCh37 | NA | NA | NA | NA | NA | 1 | 18145 | 0 | NA |
| eqtl-a-ENSG00000184185 | ENSG00000184185 | NA | public | 2018 | Vosa U | NA | Males and Females | NA | European | NA | 7444 | HG19/GRCh37 | NA | NA | NA | NA | NA | 1 | 14694 | 0 | NA |
| eqtl-a-ENSG00000011021 | ENSG00000011021 | NA | public | 2018 | Vosa U | NA | Males and Females | NA | European | NA | 31684 | HG19/GRCh37 | NA | NA | NA | NA | NA | 1 | 19291 | 0 | NA |
| eqtl-a-ENSG00000186815 | ENSG00000186815 | NA | public | 2018 | Vosa U | NA | Males and Females | NA | European | NA | 31684 | HG19/GRCh37 | NA | NA | NA | NA | NA | 1 | 18311 | 0 | NA |
| eqtl-a-ENSG00000153253 | ENSG00000153253 | NA | public | 2018 | Vosa U | NA | Males and Females | NA | European | NA | 31684 | HG19/GRCh37 | NA | NA | NA | NA | NA | 1 | 17369 | 0 | NA |
| eqtl-a-ENSG00000171385 | ENSG00000171385 | NA | public | 2018 | Vosa U | NA | Males and Females | NA | European | NA | 30551 | HG19/GRCh37 | NA | NA | NA | NA | NA | 1 | 18749 | 0 | NA |
| eqtl-a-ENSG00000135124 | ENSG00000135124 | NA | public | 2018 | Vosa U | NA | Males and Females | NA | European | NA | 31684 | HG19/GRCh37 | NA | NA | NA | NA | NA | 1 | 18258 | 0 | NA |
| eqtl-a-ENSG00000165272 | ENSG00000165272 | NA | public | 2018 | Vosa U | NA | Males and Females | NA | European | NA | 31684 | HG19/GRCh37 | NA | NA | NA | NA | NA | 1 | 18286 | 0 | NA |
| eqtl-a-ENSG00000111886 | ENSG00000111886 | NA | public | 2018 | Vosa U | NA | Males and Females | NA | European | NA | 31684 | HG19/GRCh37 | NA | NA | NA | NA | NA | 1 | 17934 | 0 | NA |
| eqtl-a-ENSG00000150995 | ENSG00000150995 | NA | public | 2018 | Vosa U | NA | Males and Females | NA | European | NA | 31684 | HG19/GRCh37 | NA | NA | NA | NA | NA | 1 | 21077 | 0 | NA |
| eqtl-a-ENSG00000169432 | ENSG00000169432 | NA | public | 2018 | Vosa U | NA | Males and Females | NA | European | NA | 31470 | HG19/GRCh37 | NA | NA | NA | NA | NA | 1 | 18630 | 0 | NA |
| eqtl-a-ENSG00000070985 | ENSG00000070985 | NA | public | 2018 | Vosa U | NA | Males and Females | NA | European | NA | 30859 | HG19/GRCh37 | NA | NA | NA | NA | NA | 1 | 20459 | 0 | NA |
| eqtl-a-ENSG00000111319 | ENSG00000111319 | NA | public | 2018 | Vosa U | NA | Males and Females | NA | European | NA | 31684 | HG19/GRCh37 | NA | NA | NA | NA | NA | 1 | 18443 | 0 | NA |
| eqtl-a-ENSG00000173338 | ENSG00000173338 | NA | public | 2018 | Vosa U | NA | Males and Females | NA | European | NA | 31684 | HG19/GRCh37 | NA | NA | NA | NA | NA | 1 | 16299 | 0 | NA |
| eqtl-a-ENSG00000196811 | ENSG00000196811 | NA | public | 2018 | Vosa U | NA | Males and Females | NA | European | NA | 30013 | HG19/GRCh37 | NA | NA | NA | NA | NA | 1 | 18095 | 0 | NA |
| eqtl-a-ENSG00000124134 | ENSG00000124134 | NA | public | 2018 | Vosa U | NA | Males and Females | NA | European | NA | 31684 | HG19/GRCh37 | NA | NA | NA | NA | NA | 1 | 18387 | 0 | NA |
| eqtl-a-ENSG00000108556 | ENSG00000108556 | NA | public | 2018 | Vosa U | NA | Males and Females | NA | European | NA | 31684 | HG19/GRCh37 | NA | NA | NA | NA | NA | 1 | 20524 | 0 | NA |
| eqtl-a-ENSG00000103569 | ENSG00000103569 | NA | public | 2018 | Vosa U | NA | Males and Females | NA | European | NA | 31684 | HG19/GRCh37 | NA | NA | NA | NA | NA | 1 | 19699 | 0 | NA |
| eqtl-a-ENSG00000099822 | ENSG00000099822 | NA | public | 2018 | Vosa U | NA | Males and Females | NA | European | NA | 14263 | HG19/GRCh37 | NA | NA | NA | NA | NA | 1 | 17905 | 0 | NA |
| eqtl-a-ENSG00000073464 | ENSG00000073464 | NA | public | 2018 | Vosa U | NA | Males and Females | NA | European | NA | 9188 | HG19/GRCh37 | NA | NA | NA | NA | NA | 1 | 10125 | 0 | NA |
| eqtl-a-ENSG00000167723 | ENSG00000167723 | NA | public | 2018 | Vosa U | NA | Males and Females | NA | European | NA | 30765 | HG19/GRCh37 | NA | NA | NA | NA | NA | 1 | 20394 | 0 | NA |
| eqtl-a-ENSG00000136802 | ENSG00000136802 | NA | public | 2018 | Vosa U | NA | Males and Females | NA | European | NA | 31684 | HG19/GRCh37 | NA | NA | NA | NA | NA | 1 | 18726 | 0 | NA |
| eqtl-a-ENSG00000131398 | ENSG00000131398 | NA | public | 2018 | Vosa U | NA | Males and Females | NA | European | NA | 31684 | HG19/GRCh37 | NA | NA | NA | NA | NA | 1 | 19126 | 0 | NA |
| eqtl-a-ENSG00000188037 | ENSG00000188037 | NA | public | 2018 | Vosa U | NA | Males and Females | NA | European | NA | 31684 | HG19/GRCh37 | NA | NA | NA | NA | NA | 1 | 15983 | 0 | NA |
| eqtl-a-ENSG00000039987 | ENSG00000039987 | NA | public | 2018 | Vosa U | NA | Males and Females | NA | European | NA | 29602 | HG19/GRCh37 | NA | NA | NA | NA | NA | 1 | 16948 | 0 | NA |
| eqtl-a-ENSG00000123104 | ENSG00000123104 | NA | public | 2018 | Vosa U | NA | Males and Females | NA | European | NA | 31684 | HG19/GRCh37 | NA | NA | NA | NA | NA | 1 | 19101 | 0 | NA |
| eqtl-a-ENSG00000165995 | ENSG00000165995 | NA | public | 2018 | Vosa U | NA | Males and Females | NA | European | NA | 30962 | HG19/GRCh37 | NA | NA | NA | NA | NA | 1 | 19169 | 0 | NA |
| eqtl-a-ENSG00000156113 | ENSG00000156113 | NA | public | 2018 | Vosa U | NA | Males and Females | NA | European | NA | 31086 | HG19/GRCh37 | NA | NA | NA | NA | NA | 1 | 18298 | 0 | NA |
| eqtl-a-ENSG00000105464 | ENSG00000105464 | NA | public | 2018 | Vosa U | NA | Males and Females | NA | European | NA | 31684 | HG19/GRCh37 | NA | NA | NA | NA | NA | 1 | 19797 | 0 | NA |
| eqtl-a-ENSG00000116396 | ENSG00000116396 | NA | public | 2018 | Vosa U | NA | Males and Females | NA | European | NA | 31684 | HG19/GRCh37 | NA | NA | NA | NA | NA | 1 | 17889 | 0 | NA |
| eqtl-a-ENSG00000196689 | ENSG00000196689 | NA | public | 2018 | Vosa U | NA | Males and Females | NA | European | NA | 30351 | HG19/GRCh37 | NA | NA | NA | NA | NA | 1 | 20354 | 0 | NA |
| eqtl-a-ENSG00000090674 | ENSG00000090674 | NA | public | 2018 | Vosa U | NA | Males and Females | NA | European | NA | 31684 | HG19/GRCh37 | NA | NA | NA | NA | NA | 1 | 20978 | 0 | NA |
| eqtl-a-ENSG00000107593 | ENSG00000107593 | NA | public | 2018 | Vosa U | NA | Males and Females | NA | European | NA | 31684 | HG19/GRCh37 | NA | NA | NA | NA | NA | 1 | 17391 | 0 | NA |
| eqtl-a-ENSG00000092439 | ENSG00000092439 | NA | public | 2018 | Vosa U | NA | Males and Females | NA | European | NA | 31430 | HG19/GRCh37 | NA | NA | NA | NA | NA | 1 | 18038 | 0 | NA |
| eqtl-a-ENSG00000102001 | ENSG00000102001 | NA | public | 2018 | Vosa U | NA | Males and Females | NA | European | NA | 9188 | HG19/GRCh37 | NA | NA | NA | NA | NA | 1 | 10125 | 0 | NA |
| eqtl-a-ENSG00000151062 | ENSG00000151062 | NA | public | 2018 | Vosa U | NA | Males and Females | NA | European | NA | 31470 | HG19/GRCh37 | NA | NA | NA | NA | NA | 1 | 19004 | 0 | NA |
| eqtl-a-ENSG00000164626 | ENSG00000164626 | NA | public | 2018 | Vosa U | NA | Males and Females | NA | European | NA | 31684 | HG19/GRCh37 | NA | NA | NA | NA | NA | 1 | 19069 | 0 | NA |
| eqtl-a-ENSG00000189280 | ENSG00000189280 | NA | public | 2018 | Vosa U | NA | Males and Females | NA | European | NA | 29094 | HG19/GRCh37 | NA | NA | NA | NA | NA | 1 | 17148 | 0 | NA |
| eqtl-a-ENSG00000197147 | ENSG00000197147 | NA | public | 2018 | Vosa U | NA | Males and Females | NA | European | NA | 31256 | HG19/GRCh37 | NA | NA | NA | NA | NA | 1 | 17308 | 0 | NA |
| eqtl-a-ENSG00000157388 | ENSG00000157388 | NA | public | 2018 | Vosa U | NA | Males and Females | NA | European | NA | 31684 | HG19/GRCh37 | NA | NA | NA | NA | NA | 1 | 18821 | 0 | NA |
| eqtl-a-ENSG00000132259 | ENSG00000132259 | NA | public | 2018 | Vosa U | NA | Males and Females | NA | European | NA | 26395 | HG19/GRCh37 | NA | NA | NA | NA | NA | 1 | 20902 | 0 | NA |
| eqtl-a-ENSG00000196218 | ENSG00000196218 | NA | public | 2018 | Vosa U | NA | Males and Females | NA | European | NA | 31470 | HG19/GRCh37 | NA | NA | NA | NA | NA | 1 | 18368 | 0 | NA |
| eqtl-a-ENSG00000026559 | ENSG00000026559 | NA | public | 2018 | Vosa U | NA | Males and Females | NA | European | NA | 31470 | HG19/GRCh37 | NA | NA | NA | NA | NA | 1 | 19337 | 0 | NA |
| eqtl-a-ENSG00000155962 | ENSG00000155962 | NA | public | 2018 | Vosa U | NA | Males and Females | NA | European | NA | 9188 | HG19/GRCh37 | NA | NA | NA | NA | NA | 1 | 10125 | 0 | NA |
| eqtl-a-ENSG00000123700 | ENSG00000123700 | NA | public | 2018 | Vosa U | NA | Males and Females | NA | European | NA | 31684 | HG19/GRCh37 | NA | NA | NA | NA | NA | 1 | 17995 | 0 | NA |
| eqtl-a-ENSG00000196876 | ENSG00000196876 | NA | public | 2018 | Vosa U | NA | Males and Females | NA | European | NA | 31470 | HG19/GRCh37 | NA | NA | NA | NA | NA | 1 | 18638 | 0 | NA |
| eqtl-a-ENSG00000129749 | ENSG00000129749 | NA | public | 2018 | Vosa U | NA | Males and Females | NA | European | NA | 31470 | HG19/GRCh37 | NA | NA | NA | NA | NA | 1 | 21013 | 0 | NA |
| eqtl-a-ENSG00000110881 | ENSG00000110881 | NA | public | 2018 | Vosa U | NA | Males and Females | NA | European | NA | 25690 | HG19/GRCh37 | NA | NA | NA | NA | NA | 1 | 16307 | 0 | NA |
| eqtl-a-ENSG00000163873 | ENSG00000163873 | NA | public | 2018 | Vosa U | NA | Males and Females | NA | European | NA | 30337 | HG19/GRCh37 | NA | NA | NA | NA | NA | 1 | 17540 | 0 | NA |
| eqtl-a-ENSG00000149403 | ENSG00000149403 | NA | public | 2018 | Vosa U | NA | Males and Females | NA | European | NA | 31684 | HG19/GRCh37 | NA | NA | NA | NA | NA | 1 | 18290 | 0 | NA |
| eqtl-a-ENSG00000142959 | ENSG00000142959 | NA | public | 2018 | Vosa U | NA | Males and Females | NA | European | NA | 31684 | HG19/GRCh37 | NA | NA | NA | NA | NA | 1 | 17174 | 0 | NA |
| eqtl-a-ENSG00000096433 | ENSG00000096433 | NA | public | 2018 | Vosa U | NA | Males and Females | NA | European | NA | 31684 | HG19/GRCh37 | NA | NA | NA | NA | NA | 1 | 26381 | 0 | NA |
| eqtl-a-ENSG00000171714 | ENSG00000171714 | NA | public | 2018 | Vosa U | NA | Males and Females | NA | European | NA | 31684 | HG19/GRCh37 | NA | NA | NA | NA | NA | 1 | 20969 | 0 | NA |
| eqtl-a-ENSG00000135519 | ENSG00000135519 | NA | public | 2018 | Vosa U | NA | Males and Females | NA | European | NA | 31684 | HG19/GRCh37 | NA | NA | NA | NA | NA | 1 | 16284 | 0 | NA |
| eqtl-a-ENSG00000178342 | ENSG00000178342 | NA | public | 2018 | Vosa U | NA | Males and Females | NA | European | NA | 31684 | HG19/GRCh37 | NA | NA | NA | NA | NA | 1 | 17236 | 0 | NA |
| eqtl-a-ENSG00000177119 | ENSG00000177119 | NA | public | 2018 | Vosa U | NA | Males and Females | NA | European | NA | 26609 | HG19/GRCh37 | NA | NA | NA | NA | NA | 1 | 17613 | 0 | NA |
| eqtl-a-ENSG00000159212 | ENSG00000159212 | NA | public | 2018 | Vosa U | NA | Males and Females | NA | European | NA | 31684 | HG19/GRCh37 | NA | NA | NA | NA | NA | 1 | 18894 | 0 | NA |
| eqtl-a-ENSG00000143595 | ENSG00000143595 | NA | public | 2018 | Vosa U | NA | Males and Females | NA | European | NA | 31470 | HG19/GRCh37 | NA | NA | NA | NA | NA | 1 | 16096 | 0 | NA |
| eqtl-a-ENSG00000177301 | ENSG00000177301 | NA | public | 2018 | Vosa U | NA | Males and Females | NA | European | NA | 30765 | HG19/GRCh37 | NA | NA | NA | NA | NA | 1 | 18610 | 0 | NA |
| eqtl-a-ENSG00000116032 | ENSG00000116032 | NA | public | 2018 | Vosa U | NA | Males and Females | NA | European | NA | 26609 | HG19/GRCh37 | NA | NA | NA | NA | NA | 1 | 20144 | 0 | NA |
| eqtl-a-ENSG00000047617 | ENSG00000047617 | NA | public | 2018 | Vosa U | NA | Males and Females | NA | European | NA | 23703 | HG19/GRCh37 | NA | NA | NA | NA | NA | 1 | 18735 | 0 | NA |
| eqtl-a-ENSG00000146205 | ENSG00000146205 | NA | public | 2018 | Vosa U | NA | Males and Females | NA | European | NA | 29432 | HG19/GRCh37 | NA | NA | NA | NA | NA | 1 | 20752 | 0 | NA |
| eqtl-a-ENSG00000198626 | ENSG00000198626 | NA | public | 2018 | Vosa U | NA | Males and Females | NA | European | NA | 31684 | HG19/GRCh37 | NA | NA | NA | NA | NA | 1 | 20643 | 0 | NA |
| eqtl-a-ENSG00000168356 | ENSG00000168356 | NA | public | 2018 | Vosa U | NA | Males and Females | NA | European | NA | 29094 | HG19/GRCh37 | NA | NA | NA | NA | NA | 1 | 17603 | 0 | NA |
| eqtl-a-ENSG00000103249 | ENSG00000103249 | NA | public | 2018 | Vosa U | NA | Males and Females | NA | European | NA | 31684 | HG19/GRCh37 | NA | NA | NA | NA | NA | 1 | 21348 | 0 | NA |
| eqtl-a-ENSG00000183960 | ENSG00000183960 | NA | public | 2018 | Vosa U | NA | Males and Females | NA | European | NA | 30935 | HG19/GRCh37 | NA | NA | NA | NA | NA | 1 | 18174 | 0 | NA |
| eqtl-a-ENSG00000153898 | ENSG00000153898 | NA | public | 2018 | Vosa U | NA | Males and Females | NA | European | NA | 31684 | HG19/GRCh37 | NA | NA | NA | NA | NA | 1 | 18165 | 0 | NA |
| eqtl-a-ENSG00000198515 | ENSG00000198515 | NA | public | 2018 | Vosa U | NA | Males and Females | NA | European | NA | 28345 | HG19/GRCh37 | NA | NA | NA | NA | NA | 1 | 16382 | 0 | NA |
| eqtl-a-ENSG00000109572 | ENSG00000109572 | NA | public | 2018 | Vosa U | NA | Males and Females | NA | European | NA | 31684 | HG19/GRCh37 | NA | NA | NA | NA | NA | 1 | 18755 | 0 | NA |
| eqtl-a-ENSG00000177807 | ENSG00000177807 | NA | public | 2018 | Vosa U | NA | Males and Females | NA | European | NA | 31684 | HG19/GRCh37 | NA | NA | NA | NA | NA | 1 | 18612 | 0 | NA |
| eqtl-a-ENSG00000053918 | ENSG00000053918 | NA | public | 2018 | Vosa U | NA | Males and Females | NA | European | NA | 31470 | HG19/GRCh37 | NA | NA | NA | NA | NA | 1 | 20704 | 0 | NA |
| eqtl-a-ENSG00000100433 | ENSG00000100433 | NA | public | 2018 | Vosa U | NA | Males and Females | NA | European | NA | 31346 | HG19/GRCh37 | NA | NA | NA | NA | NA | 1 | 18058 | 0 | NA |
| eqtl-a-ENSG00000169583 | ENSG00000169583 | NA | public | 2018 | Vosa U | NA | Males and Females | NA | European | NA | 31684 | HG19/GRCh37 | NA | NA | NA | NA | NA | 1 | 18471 | 0 | NA |
| eqtl-a-ENSG00000121743 | ENSG00000121743 | NA | public | 2018 | Vosa U | NA | Males and Females | NA | European | NA | 31256 | HG19/GRCh37 | NA | NA | NA | NA | NA | 1 | 20219 | 0 | NA |
| eqtl-a-ENSG00000133962 | ENSG00000133962 | NA | public | 2018 | Vosa U | NA | Males and Females | NA | European | NA | 26609 | HG19/GRCh37 | NA | NA | NA | NA | NA | 1 | 19493 | 0 | NA |
| eqtl-a-ENSG00000137672 | ENSG00000137672 | NA | public | 2018 | Vosa U | NA | Males and Females | NA | European | NA | 31684 | HG19/GRCh37 | NA | NA | NA | NA | NA | 1 | 19317 | 0 | NA |
| eqtl-a-ENSG00000176884 | ENSG00000176884 | NA | public | 2018 | Vosa U | NA | Males and Females | NA | European | NA | 29218 | HG19/GRCh37 | NA | NA | NA | NA | NA | 1 | 18128 | 0 | NA |
| eqtl-a-ENSG00000055118 | ENSG00000055118 | NA | public | 2018 | Vosa U | NA | Males and Females | NA | European | NA | 31684 | HG19/GRCh37 | NA | NA | NA | NA | NA | 1 | 20126 | 0 | NA |
| eqtl-a-ENSG00000158445 | ENSG00000158445 | NA | public | 2018 | Vosa U | NA | Males and Females | NA | European | NA | 12995 | HG19/GRCh37 | NA | NA | NA | NA | NA | 1 | 17975 | 0 | NA |
| eqtl-a-ENSG00000100346 | ENSG00000100346 | NA | public | 2018 | Vosa U | NA | Males and Females | NA | European | NA | 31086 | HG19/GRCh37 | NA | NA | NA | NA | NA | 1 | 17194 | 0 | NA |
| eqtl-a-ENSG00000171365 | ENSG00000171365 | NA | public | 2018 | Vosa U | NA | Males and Females | NA | European | NA | 9188 | HG19/GRCh37 | NA | NA | NA | NA | NA | 1 | 10125 | 0 | NA |
| eqtl-a-ENSG00000107147 | ENSG00000107147 | NA | public | 2018 | Vosa U | NA | Males and Females | NA | European | NA | 26609 | HG19/GRCh37 | NA | NA | NA | NA | NA | 1 | 20906 | 0 | NA |
| eqtl-a-ENSG00000166762 | ENSG00000166762 | NA | public | 2018 | Vosa U | NA | Males and Females | NA | European | NA | 14263 | HG19/GRCh37 | NA | NA | NA | NA | NA | 1 | 14005 | 0 | NA |
| eqtl-a-ENSG00000122986 | ENSG00000122986 | NA | public | 2018 | Vosa U | NA | Males and Females | NA | European | NA | 30935 | HG19/GRCh37 | NA | NA | NA | NA | NA | 1 | 16340 | 0 | NA |
| eqtl-a-ENSG00000169504 | ENSG00000169504 | NA | public | 2018 | Vosa U | NA | Males and Females | NA | European | NA | 31430 | HG19/GRCh37 | NA | NA | NA | NA | NA | 1 | 17770 | 0 | NA |
| eqtl-a-ENSG00000130529 | ENSG00000130529 | NA | public | 2018 | Vosa U | NA | Males and Females | NA | European | NA | 31684 | HG19/GRCh37 | NA | NA | NA | NA | NA | 1 | 18889 | 0 | NA |
| eqtl-a-ENSG00000102057 | ENSG00000102057 | NA | public | 2018 | Vosa U | NA | Males and Females | NA | European | NA | 9188 | HG19/GRCh37 | NA | NA | NA | NA | NA | 1 | 10125 | 0 | NA |
| eqtl-a-ENSG00000187486 | ENSG00000187486 | NA | public | 2018 | Vosa U | NA | Males and Females | NA | European | NA | 14263 | HG19/GRCh37 | NA | NA | NA | NA | NA | 1 | 17121 | 0 | NA |
| eqtl-a-ENSG00000105711 | ENSG00000105711 | NA | public | 2018 | Vosa U | NA | Males and Females | NA | European | NA | 31684 | HG19/GRCh37 | NA | NA | NA | NA | NA | 1 | 18732 | 0 | NA |
| eqtl-a-ENSG00000213719 | ENSG00000213719 | NA | public | 2018 | Vosa U | NA | Males and Females | NA | European | NA | 14263 | HG19/GRCh37 | NA | NA | NA | NA | NA | 1 | 41180 | 0 | NA |
| eqtl-a-ENSG00000177272 | ENSG00000177272 | NA | public | 2018 | Vosa U | NA | Males and Females | NA | European | NA | 31684 | HG19/GRCh37 | NA | NA | NA | NA | NA | 1 | 18667 | 0 | NA |
| eqtl-a-ENSG00000157445 | ENSG00000157445 | NA | public | 2018 | Vosa U | NA | Males and Females | NA | European | NA | 31684 | HG19/GRCh37 | NA | NA | NA | NA | NA | 1 | 18914 | 0 | NA |
