# Supplementary material for: Using eQTL Mendelian randomization and transcriptomic analysis to identify the relationship between ion channel genes and intracranial aneurysmal subarachnoid hemorrhage
Source: Medicine (Baltimore). 2025 May 16;104(20):e42457. doi: 10.1097/MD.0000000000042457 (PMC12091597; doi:10.1097/MD.0000000000042457)
Supplement: Supplementary file 3 [file medi-104-e42457-s003.docx]

| **Table S3 Mendelian Randomization Analysis Using Five Distinct Algorithms** | | | | | | | | |
| --- | --- | --- | --- | --- | --- | --- | --- | --- |
| exposure | outcome | Nsnp | Method | OR | Beta | Beta_lci95 | Beta_uci95 | Pvalue |
| ANO6 | aSAH | 6 | Inverse variance weighted (fixed effects) | 0.728(0.533~0.993) | -0.318 | -0.629 | -0.007 | 0.045 |
| ANO6 | aSAH | 6 | Simple median | 0.687(0.471~1.004) | -0.375 | -0.754 | 0.004 | 0.052 |
| ANO6 | aSAH | 6 | Weighted median | 0.757(0.517~1.108) | -0.279 | -0.659 | 0.102 | 0.152 |
| ANO6 | aSAH | 6 | Simple mode | 0.735(0.4~1.35) | -0.307 | -0.915 | 0.300 | 0.367 |
| ANO6 | aSAH | 6 | Weighted mode | 0.83(0.504~1.369) | -0.186 | -0.686 | 0.314 | 0.499 |
| CACNA2D3 | aSAH | 14 | Inverse variance weighted (multiplicative random effects) | 1.245(1.008~1.537) | 0.219 | 0.008 | 0.430 | 0.042 |
| CACNA2D3 | aSAH | 14 | Simple median | 1.43(1.093~1.87) | 0.358 | 0.089 | 0.626 | 0.009 |
| CACNA2D3 | aSAH | 14 | Weighted median | 1.197(0.99~1.447) | 0.180 | -0.010 | 0.370 | 0.063 |
| CACNA2D3 | aSAH | 14 | Simple mode | 1.345(0.917~1.974) | 0.296 | -0.087 | 0.680 | 0.154 |
| CACNA2D3 | aSAH | 14 | Weighted mode | 1.19(0.973~1.456) | 0.174 | -0.028 | 0.376 | 0.115 |
