# Supplementary material for: Using eQTL Mendelian randomization and transcriptomic analysis to identify the relationship between ion channel genes and intracranial aneurysmal subarachnoid hemorrhage
Source: Medicine (Baltimore). 2025 May 16;104(20):e42457. doi: 10.1097/MD.0000000000042457 (PMC12091597; doi:10.1097/MD.0000000000042457)
Supplement: Supplementary file 4 [file medi-104-e42457-s004.docx]

| **Table S4 Assessment of Heterogeneity in MR Analysis** | | | | | |
| --- | --- | --- | --- | --- | --- |
| exposure | outcome | method | Q | Q_df | Q_pval |
| ANO6 | aSAH | MR Egger | 1.322 | 4 | 0.858 |
| ANO6 | aSAH | Inverse variance weighted | 2.506 | 5 | 0.776 |
| CACNA2D3 | aSAH | MR Egger | 26.597 | 12 | 0.009 |
| CACNA2D3 | aSAH | Inverse variance weighted | 29.288 | 13 | 0.006 |
