# Supplementary material for: Using eQTL Mendelian randomization and transcriptomic analysis to identify the relationship between ion channel genes and intracranial aneurysmal subarachnoid hemorrhage
Source: Medicine (Baltimore). 2025 May 16;104(20):e42457. doi: 10.1097/MD.0000000000042457 (PMC12091597; doi:10.1097/MD.0000000000042457)
Supplement: Supplementary file 5 [file medi-104-e42457-s005.docx]

| **Table S5 IVW Model Selection and Pleiotropy Assessment for CACNA2D3 and ANO6** | | | | |
| --- | --- | --- | --- | --- |
| exposure | outcome | egger_intercept | se | pval |
| ANO6 | aSAH | -0.051 | 0.047 | 0.338 |
| CACNA2D3 | aSAH | 0.030 | 0.027 | 0.292 |
|  |  |  |  |  |
| exposure | outcome | MR_PRESSO_P | |  |
| ANO6 | aSAH | 0.781 |  |  |
| CACNA2D3 | aSAH | 0.54 |  |  |
