# Supplementary material for: Using eQTL Mendelian randomization and transcriptomic analysis to identify the relationship between ion channel genes and intracranial aneurysmal subarachnoid hemorrhage
Source: Medicine (Baltimore). 2025 May 16;104(20):e42457. doi: 10.1097/MD.0000000000042457 (PMC12091597; doi:10.1097/MD.0000000000042457)
Supplement: Supplementary file 6 [file medi-104-e42457-s006.docx]

| **Table S6** **Steiger Directionality Test for Exposure-Outcome Causal Inference** | | | | | |
| --- | --- | --- | --- | --- | --- |
| exposure | outcome | snp_r2.exposure | snp_r2.outcome | correct_causal_direction | steiger_pval |
| ANO6 | aSAH | 0.009 | 8.43E-05 | TRUE | 3.93E-34 |
| CACNA2D3 | aSAH | 0.036 | 0.000499 | TRUE | 1.17E-144 |
